# Insulin-like growth factors and cancer.

**DOI:** 10.1038/bjc.1992.65

**Published:** 1992-03

**Authors:** V. M. Macaulay

**Affiliations:** Section of Cell and Molecular Biology, Chester Beatty Laboratories, Institute of Cancer Research, London, UK.


Br. J. Cancer (1992), 65, 311-320 © Macmillan Press Ltd., 1992

REVIEW

### Insulin-like growth factors and cancer

V.M. Macaulay

*Section of Cell and Molecular Biology, Chester Beatty Laboratories, Institute of Cancer Research, Fulham Road, London SW3 6JJ, UK.*

The insulin-like growth factors (IGFs), also known as somatomedins, have been identified as a result of three separate lines of research carried out over the last 30 years (Van Wyk & Underwood, 1978). First, IGFs promote incorporation of $^{35}$S-sulphate into cartilage, hence 'sulphation' factor (Salmon & Daughaday, 1957). Secondly, they mediate the mitogenic activity of serum (Pierson & Temin, 1972) and medium conditioned by rat hepatocytes (multiplication stimulating activity, MSA; Dulak & Temin, 1973). Thirdy, IGFs have insulin-like activity which is not inhibited by anti-insulin antibodies (non-suppressive insulin-like activity, NSILA; Froesch *et al.*, 1963). Sequence analysis revealed that these functions are subserved by two main peptide: IGF-I, also known as somatomedin-C (Klapper *et al.*, 1983) and IGF-II, of which the rat form is MSA (Rinderknecht & Humbel, 1978; Marquardt *et al.*, 1981). The IGF terminology is now preferred as there is no somatomedin designation for IGF-II (Daughaday *et al.*, 1987).

IGF-I (70 residues, MW 7649) and IGF-II (67 residues, MW 7471) are single chain peptides with around 70% sequence homology, and 50% homology with pro-insulin. Mature IGFs have A and B domains where the homology with proinsulin is highest, a C-peptide domain which has no sequence homology with proinsulin, and a carboxyterminal D domain (Daughaday & Rotwein, 1989). The IGF-I gene is located on chromosome 12q, and the IGF-II gene is on chromosome 11p, contiguous with the insulin gene (Barreca & Minuto, 1989). In the mouse, the IGF-II gene is imprinted, that is, there is a difference in expression between the maternal and paternal genes. Specifically, it is the paternal IGF-II gene which is active (Willison, 1991).

IGF-I is synthesised by the liver and also by other viscera including kidney and lung (D'Ercole *et al.*, 1984). Hepatic synthesis, which largely determines serum levels, is regulated by growth hormone (GH) and also varies with liver function and nutritional status (Underwood *et al.*, 1986; Zapf & Froesch, 1986). In endocrine-sensitive tissues, IGF-I gene expression may be regulated by hormones other than GH. Notably in rat uterus, IGF-I expression is enhanced by oestrogen, and is repressed to a small extent by GH (Murphy & Friesen, 1988). *In vitro*, IGF-I is a potent mitogen for normal cells including chondrocytes and other mesenchymal derivatives (Clemmons & Van Wyk, 1981). *In vivo*, IGF-I has acute insulin-like anabolic effects on adipose tissue, muscle and liver (Zapf & Froesch, 1986; Guler *et al.*, 1987). However its most important physiological role is as the primary regulator of growth, especially of mesenchymal tissues including bone and cartilage (Schoenle *et al.*, 1982; Van Buul-Offers *et al.*, 1986; Mathews *et al.*, 1988). IGF-II has metabolic and mitogenic effects experimentally, but its physiological function is unclear. Serum concentrations are less dependent on GH, and it causes less growth promotion in hypophysectomised animals (Schoenle *et al.*, 1983). IGF-II mRNA is expressed in foetal tissues of mesenchymal origin, including kidney, liver and muscle, and also in the CNS (Han *et al.*, 1987; Brice *et al.*, 1989). The suggestion that IGF-II may be important for foetal growth is supported by the demonstration of growth deficiency in foetal mice carrying an IGF-II gene disrupted by targetting (DeChiara *et al.*, 1990).

IGFs bind to two types of cell surface IGF receptor and also cross-react with the human insulin receptor (HIR; Rechler & Nissley, 1986). The type I IGF receptor, like the HIR, is a tetrameric $\alpha2\ \beta2$ complex in which extracellular $\alpha$ (MW 130,000) and transmembrane $\beta$ (MW 90,000) subunits are linked by disulphide bonds. The $\alpha$ subunits contain the ligand binding domains, and the intracellular portion of the $\beta$ domain has tyrosine kinase activity (Massague & Czech, 1982). The $\alpha$ and $\beta$ subunits are generated by cleavage of a transmembrane precursor polypeptide (Lammers *et al.*, 1989). The type I receptor binds IGF-I with high affinity, has 2-3 times lower affinity for IGF-II and 100 times lower affinity for insulin (Czech, 1989). Sequence analysis of the type I IGF receptor and HIR shows homology which is most pronounced (84%) in the tyrosine kinase domains (Ullrich *et al.*, 1986). Both receptors can mediate acute metabolic and longer-term mitogenic effects. *In vitro* effects on glucose transport appear similar, but chimeric receptors possessing the cytoplasmic domain of the IGF receptor are 10 times more active in stimulating DNA synthesis and are more stable (Lammers *et al.*, 1989). It is this receptor which probably mediates the mitogenic effects of both IGFs and insulin (Van Wyk *et al.*, 1985). Other *in vivo* differences in the actions of IGF-I and insulin may be attributable to the tissue distribution of their receptors (Czech, 1989). The type II IGF receptor is a single chain monomer of MW 250,000. It has much higher affinity for IGF-II than IGF-I, and negligible affinity for insulin. Like the IGF-II gene, the murine IGF-II receptor gene is imprinted, but here it is the maternal gene which is active (Willison, 1991). The type II receptor is present on virtually all cell types, and in cultured cells has been shown to mediate calcium influx and synthesis of DNA and glycogen (Massague & Czech, 1982). However, it has no intrinsic kinase activity, and it is not clear what responses are mediated via this receptor *in vivo*. Unexpectedly, the human type II IGF receptor has been found to have 80% and 99% sequence homology respectively with the bovine and human mannose-6-phosphate receptor (MPR) which participates in delivery of lysosomal enzymes to the lysosome (Morgan *et al.*, 1987; Oshima *et al.*, 1988). Proteins containing mannose-6-phosphate include lysosomal enzymes such as procathepsin-D, and pro-TGF$\beta$ (Rochefort *et al.*, 1987; Czech, 1989). It now seems that these two distinct functions are indeed subserved by the same receptor molecule, suggesting that growth regulating receptors can interact both with carbohydrates and growth factors (MacDonald *et al.*, 1988; Roth, 1988).

Circulating IGFs are carried by serum binding proteins (BPs) which are synthesised mainly in the liver (Froesch & Zapf, 1985; Hossenlopp *et al.*, 1985; see Table I). Most serum IGF is carried in a 150,000 MW complex composed of an acid-labile non-binding $\alpha$ subunit and an acid-stable binding $\beta$ subunit known as IGFBP-3 (Baxter, 1988; Czech, 1989). In this form serum IGFs have a half life of 12-15 h compared with 10-12 min for the free peptides (Guler *et al.*, 1989). The

Received 19 August 1991; and in revised form 7 November 1991.

**Table I** IGF binding proteins

| *BP size* | *Designation* | *Synonyms* | *Major source* | *Comments* |
|---|---|---|---|---|
| 41.5 38.5 | IGFBP3 | BP53 | Serum | Two different glycosylated forms |
| 34 | IGFBP2 | BP31 | CSF, serum | |
| 30 | IGFBP1 | BP25 BP28 Placental protein 12 | Amniotic fluid, serum, HepG2 CM | 33% sequence homology with BP3 |
| 24 | IGFBP4 | | Serum, fibroblast CM breast CM | |

BP = binding protein; CM = conditioned medium. From Hossenlopp *et al.*, 1986; Lamson *et al.*, 1989.

large complex probably functions as a serum IGF reservoir. This is important because unlike other hormones, IGFs are not stored in cells, but are secreted as soon as they are synthesised (Holly & Wass, 1989). A small proportion of serum IGF is carried on smaller BPs of MW 24–34,000. Circulating IGF bioactivity is controlled acutely via insulin which regulates binding protein synthesis in the liver, and chronically via changes in the rate of IGF production (Holly & Wass, 1989). Small binding proteins are also present in tissues, and usually inhibit the cellular actions of IGF-I (Ritvos *et al.*, 1988; Rutanen *et al.*, 1988). Mutation or truncation of the amino terminus of IGF-I generates analogues with reduced affinity for binding proteins and enhanced biological activity despite reduced serum half-life (Cascieri *et al.*, 1988*a*; Bagley *et al.*, 1989; Ross *et al.*, 1989). For further information on the biochemistry and function of IGFs, see reviews by Barreca and Minuto (1989), Daughaday and Rotwein (1989) and Humbel (1990).

Malignant disease may be associated with inappropriate or excessive IGF activity, manifest as mitogenic or metabolic effects.

### IGFs as tumour growth factors

IGFs are increasingly recognised as important mitogens in many tumour types, largely as a result of experimental studies *in vitro* (Daughaday, 1990). *In vivo*, tumour cell growth may be enhanced by IGFs derived from serum or tumour stroma. In addition, tumour cells with functional IGF receptors may be able to enhance their own growth by synthesis of endogenous IGFs. This process of autocrine secretion contributes to the partial autonomy and rapid growth which characterise malignant cells (Sporn & Todaro, 1980). This phenomenon has been studied particularly in the common solid tumours, including cancers of the lung, breast and gut, and the results are summarised below.

In addition to their role in promoting growth, it has recently been suggested that IGFs may play a part in neoplastic transformation and metastasis. Over-expression of the normal human type I IGF receptor in NIH 3T3 cells leads to ligand-dependent alteration in culture morphology, colony formation in soft agar and tumorigenicity in nude mice (Kaleko *et al.*, 1990). In cultured human melanoma cells, the type I receptor has been shown to mediate a highly potent motility response to IGFs and insulin, an effect which could enhance the potential for local and distant spread (Stracke *et al.*, 1989). IGFs and insulin also stimulate motility in human breast, bladder and ovarian cancer cell lines, and the concentrations required for optimal migration appear lower than for maximal growth stimulation (Kohn *et al.*, 1990).

### *Lung cancer*

Immunoreactive IGF-I is produced *in vitro* by human foetal lung explants and cultured alveolar macrophages (Snyder & D'Ercole, 1987; Rom *et al.*, 1988). Dot and Northern blot analyses show expression of IGF-I and IGF-II throughout lung organogenesis (Davenport *et al.*, 1988). IGF-I stimulates collagen formation by embryonic lung fibroblasts (Goldstein *et al.*, 1989) and growth of normal human bronchial epithelial cells (Seigfried, 1989). Thus IGFs seem to be important in lung development, and are also implicated in growth regulation of lung tumours.

Primary lung tumours possess IGF-I binding sites as shown by autoradiography, with the highest density of receptors in squamous cancers and small cell lung cancer (SCLC; Shigematsu *et al.*, 1990). Iodinated ligand binding studies on cultured SCLC cells demonstrate two classes of IGF-I binding site of high ($K_d$ 0.1–1.1 nM) and lower (3–4 nM) affinity (Nakanishi *et al.*, 1988; Macaulay *et al.*, 1990). These receptors are functional, because exogenous IGF-I causes a mitogenic response in SCLC cells (Jaques *et al.*, 1988; Nakanishi *et al.*, 1988; Macaulay *et al.*, 1988*a* and 1990) and non-SCLC (Siegfried, 1989). Immunoreactive IGF-I is detectable in primary lung tumour tissue including non-SCLC (squamous and adenocarcinoma) and SCLC, at higher levels than in normal lung (Minuto *et al.*, 1986; Macaulay *et al.*, 1988*a*). Immunohistochemistry shows strong staining for IGF-I especially in cases of squamous lung cancer (Shigimatsu *et al.*, 1990). Immunoreactive IGF-I is also detectable in extracts of cultured SCLC and non-SCLC cells and their conditioned media (Jaques *et al.*, 1988; Macaulay *et al.*, 1988*a* and 1990, Siegfried, 1989). Western blot analysis shows a 16,000 MW band consistent with an incompletely processed IGF-I precursor (Nakanishi *et al.*, 1988). However, IGF-I levels are not raised in the serum of lung cancer patients and levels are unrelated to bulk of disease or response to treatment. Thus IGF-I is not a marker for disease activity, presumably reflecting the relatively small contribution of the tumour compared with hepatic IGF-I production (Macaulay *et al.*, 1988*b*). Indeed circulating IGF-I levels may be low in association with poor nutritional status or abnormal liver function (Minuto *et al.*, 1986). In addition to IGF-I-like peptides, lung cancer cells can also synthesise IGF BPs. Cross-linking studies with $^{125}$I-IGF-I or -II show that SCLC conditioned medium contains IGF BPs with MW of 24–32,000 (Jaques *et al.*, 1989). Low MW BPs (25–30,000) are elevated in the serum of lung cancer patients compared with normal controls (Reeve *et al.*, 1990).

Thus there is good evidence that lung cancer cells produce IGF-I and IGF BPs, express IGF binding sites and exhibit a mitogenic response to exogenous IGF-I, suggesting that IGF-I can function as an autocrine growth factor for lung cancer.

### *Breast cancer*

Virtually all cultured breast cancer cell lines and fresh tumour biopsies express receptors for IGFs I and II and insulin. $^{125}$I-IGF-I binding studies show a single class of site with affinity for IGF-I (Kd 0.5–4 nM) which is five times greater than for IGF-II and 10–100 times greater than for insulin. IGF-I binding to primary and mestastatic human breast tumours is increased significantly compared with normal breast tissue. Cross-linking studies show a 130,000 band, presumably the alpha subunit of the type I receptor. In low serum or serum-free medium, growth of cultured breast cancer cells is stimulated by IGF-I 5 nM, which generates a greater response than that to optimal concentrations of oestradiol ($E_2$) or epidermal growth factor (EGF). Higher concentrations of IGF-II and insulin are required to produce a similar effect, supporting the suggestion that IGFs and insulin exert their mitogenic effects via the type I IGF receptor (Furlanetto & DiCarlo, 1984; Pollak *et al.*, 1988; Peyrat *et al.*, 1988; Foekens *et al.*, 1989*a*; Cullen *et al.*, 1990; Osborne & Arteaga, 1990).

Cultured cells and fresh tumour specimens also express IGF-I-like activity and levels are 2–10 times higher in oestrogen receptor (ER) negative than in ER positive cell lines (Huff *et al.*, 1986; Foekens *et al.*, 1989*a*). Northern analysis using a cDNA probe to IGF-I reveals a pattern of multiple cross-hybridising mRNA bands in breast cancer cell lines.

The pattern is more complex than in normal human liver (Rotwein, 1986), and is very similar to human foetal tissues (Han *et al.*, 1987), suggesting that breast cancer cells show a foetal-like IGF mRNA pattern. IGF-II mRNA has also been detected at low abundance in cultured human breast cancer cells (Freed & Herington, 1989). Using an antisense RNA probe which includes the entire coding region of the IGF-IA precursor in an RNase protection assay, Yee *et al.* (1989*a*) detected expression of IGF-I mRNA in fresh breast cancer tumour samples. However *in situ* hybridisation showed that positivity is confined to stromal cells, with no detectable IGF-I mRNA in normal or malignant epithelial cells. It is possible that the neoplastic cells are responsive to IGF-I secreted by adjacent stroma, suggesting paracrine function for IGF-I here. Similarly, this group failed to detect authentic IGF-I mRNA in breast cancer cell lines. It was suggested that the immunoreactive IGF-I detectable in cultured breast cancer cells and their conditioned media is not therefore authentic IGF-I from mRNA transcribed from the coding exons of the IGF-I gene. It could be an IGF-I related protein, or IGF BP(s) which are known to interfere in IGF-I radioimmunoassays (Yee *et al.*, 1989*a*). Radioimmunoassay, Western ligand blot and Northern analysis show that breast cancer cell lines produce BPs of MW 24–40,000, compatible with IGBPs 1, 2 and 3. In ER positive cells, IGFBP mRNA may be regulated by $E_2$ (DeLeon *et al.*, 1989; Yee *et al.*, 1989*b*; Yee *et al.*, 1991).

Regulation of IGF and IGF receptor expression in breast cancer is further complicated by the fact that cells synthesise other growth factors including TGFα and β, PDGF and pro-cathepsin D (52 k protein), and some cell lines and tumours are hormone sensitive (Osborne & Arteaga, 1990). Oestradiol-induced growth of breast cancer cells *in vitro* may be associated with increased secretion of IGF-I-like peptides (Huff *et al.*, 1988), but other reports have shown little or no IGF response to $E_2$ (Dickson *et al.*, 1986; Freed & Herington, 1989). IGF-I synthesis is regulated at a post-transcriptional step, and is induced by EGF and TGFα, and inhibited by TGFβ, dexamethasone and tamoxifen. Transfection of hormone-sensitive MCF-7 cells with the v-Harvey-*ras* oncogene leads to increased secretion of immunoreactive IGF-I and partial autonomy from exongenous IGF-I, oestrogen and anti-oestrogens (Dickson *et al.*, 1987; Huff *et al.*, 1988). Breast cancer cell growth and immunoreactive IGF-I secretion are unaffected by growth hormone, basic FGF, PDGF or prolactin, indicating that IGF-I regulation here occurs by mechanisms which differ from those in human fibroblasts (Huff *et al.*, 1988). It is possible that PDGF secreted locally by breast cancer cells could stimulate synthesis by stromal fibroblasts of IGF-I, which in turn could enhance the growth of the breast cancer cells (Yee *et al.*, 1989*a*). Further support for a regulatory link between IGFs and hormones comes from studies of fresh breast cancer tumour where ER expression is positively correlated with expression of type I IGF receptor, and inversely correlated with levels of IGF-I immunoreactivity (Pekonen *et al.*, 1988; Peyrat *et al.*, 1988; Foekens *et al.*, 1989*a*). Unlike ER and EGF receptor expression, detection of IGF receptor is not of prognostic significance (Foekens *et al.*, 1989*b*). IGF-I can at least partially substitute for oestrogen in stimulating breast cancer proliferation *in vitro* and *in vivo* and may be an important mediator of oestrogenic effects in breast cancer (Dickson *et al.*, 1986). However, IGF-I can synergise with $E_2$ in enhancing progesterone receptor (PR) synthesis by ER positive MCF-7 cells, suggesting utilisation of different pathways (Katzenellenbogen & Normon, 1990). IGFs may also be involved in regulation of cathepsin D synthesis by breast cancer cells (Cavailles *et al.*, 1989).

Thus there is continuing debate about the role of IGFs as 'oestromedins', given the conflicting evidence regarding the effect of $E_2$ on endogenous IGF-I-like peptide production, the doubt as to whether breast cancer cells produce authentic IGF-I, and the synergy between IGF-I and $E_2$ on PR synthesis (Freed & Herington, 1989; Yee *et al.*, 1989*a*; Katzenellenbogen & Norman, 1990).

*Other solid tumours*

IGFs are involved in the normal growth and development of viscera including the gastrointestinal tract (van Schravendijk *et al.*, 1987; Laburthe *et al.*, 1988), liver and kidney (D'Ercole *et al.*, 1984; Fagin & Melmed, 1987). They are also implicated in growth regulation of tumours derived from these tissues (see Table II). Several studies have demonstrated over-expression of IGF receptors by tumour cells compared with the corresponding normal tissues, for example in thyroid cancer (Yashiro *et al.*, 1989), hepatoma (Hartshorn *et al.*, 1989) and endometrial carcinoma (Talavera *et al.*, 1990). Some have shown over-expression of IGFs themselves, for example the study of Lambert *et al.* (1990) where IGF-II mRNA was found to be increased up to 800-fold in colorectal cancers compared with adjacent normal tissues. This group also detected a restriction fragment length polymorphism in 1/13 cases, suggesting structural modification of one IGF-II allele in the tumour compared with normal tissue.

*Embryonal tumours*

Wilms' tumour is thought to arise from embryonal kidney cells, and most specimens show elevation of IGF-II transcripts to levels comparable with those in foetal kidney (Reeve *et al.*, 1985; Scott *et al.*, 1985). Although IGF-II mRNA is over-expressed up to 30-fold, immunoreactive IGF-II is expressed at only 4–6 times higher levels than normal kidney. This suggests regulation at the translational level, the presence of non-functional mRNA, or that IGF-II is being degraded or secreted more rapidly than normal (Haselbacher *et al.*, 1987). The IGF-II gene is located on chromosome 11p, near the Wilms' tumour susceptibility gene. There is a single case report of a Wilms' tumour patient with a structural alteration in an IGF-II gene, but there is no other evidence of IGF-II gene amplification or rearrangement (Reeve *et al.*, 1985; Irminger *et al.*, 1989). Furthermore, a study of Wilms' tumour xenografts growing in nude mice showed inconsistent over-expression of IGF-II mRNA in successive passage tumours. In this model, therefore, elevation of IGF-II mRNA does not seem to be an obligatory event in Wilm's tumour progression (Little *et al.*, 1987). However the association between the IGF-II gene and Wilms' tumour has been strengthened by recent genetic analysis of patients with Beckwith-Wiedemann syndrome (BWS; Beckwith, 1963; Wiedemann, 1964). This is a rare foetal over-growth syndrome characterised by exomphalos, macroglossia, gigantism, hypoglycaemia and visceromegaly. Around 12% of these patients develop embryonal neoplasia including Wilms', hepatoblastoma and rhabdomyosarcoma. Henry *et al.* (1991) have shown that a significant proportion of BWS patients have uniparental paternal disomy for 11p15.5, that is, both copies had been inherited from the father. The disomic region includes the genes for insulin and IGF-II. Where loss of an 11p15.5 allele has occurred in BWS tumours, it is always the maternal one. If the human IGF-II gene is imprinted like the murine gene, duplication of the active paternal allele could explain the features of BWS (Henry *et al.*, 1991; Little *et al.*, 1991).

IGF-II has also been shown to stimulate the growth of cells from another embryonal tumour, neuroblastoma. *In situ* hybridisation showed that IGF-II mRNA is infrequently expressed by the tumour cells, but is detectable in all cases in non-malignant adrenal cortical and stromal cells, suggesting a paracrine role for IGF-II here (El-Badry *et al.*, 1991).

### IGFs as treatment targets

The evidence reviewed above suggests that IGFs are important determinants of tumour growth at least in experimental models. How far this applies clinically can best be judged by the extent to which tumour growth is inhibited by blocking the effects of IGFs. To date there have been only limited attempts to develop such treatments. This may be partly

**Table II** Tumour expression of IGFs and IGF receptors

| Tumour | Production of IGFs | Production of BPs | IGF receptors | Mitogenic response to IGFs | Comments | References |
|---|---|---|---|---|---|---|
| Lung | IGF-I | 24-32K | Type I | IGF-I > IGF-II, insulin | | Jaques *et al.*, 1988, 1989<br>Macaulay *et al.*, 1988*a*, 1990<br>Minuto *et al.*, 1986, 1988<br>Nakanishi *et al.*, 1988<br>Reeve *et al.*, 1990<br>Shigematsu *et al.*, 1990<br>Siegfried, 1989 |
| Breast | Authentic IGF-I production by stromal cells not tumour cells | 24-40K | Type I<br>Type II insulin | IGF-I > IGF-II, insulin | Type I receptor expression correlates with levels of ER. BP production regulated by $E_2$ | Furnaletto & DiCarlo, 1984<br>Huff *et al.*, 1986, 1988<br>Peyrat *et al.*, 1988<br>Pollak *et al.*, 1988<br>De Leon *et al.*, 1989<br>Foekens *et al.*, 1989*a,b*<br>Freed & Herington, 1989<br>Yee *et al.*, 1989*a,b*<br>Cullen *et al.*, 1990<br>Osborne & Arteaga, 1990 |
| Thyroid carcinoma | IGF-I | | Type I | | IGF-I production & IGF-I binding capacity carcinoma > normal thyroid | Minuto *et al.*, 1989<br>Yashiro *et al.*, 1989 |
| adenoma | IGF-I | | | | | Wynne-Williams *et al.*, 1989 |
| MTC | IGF-I | | Type I | IGF-I | MTC cells express IGF-II mRNA but IGF-II secretion undetectable | Okimura *et al.*, 1989<br>Suzuki *et al.*, 1989 |
| Thymoma | | | Type I + II | | | Verland & Gammeltoft, 1989 |
| Gastric | IGF-II | * | Type I + II | IGF-I<br>MSA | *IGF BPs not characterised | Whitehead *et al.*, 1989<br>Thompson *et al.*, 1990 |
| Colon | IGF-II | | Type I | IGF-I, insulin | IGF-II mRNA levels highest in distal and Duke's C lesions. IGF-I stimulates growth of high > low metastatic variant of mouse colon adenocarcinoma | Tricoli *et al.*, 1986<br>Pollak *et al.*, 1987<br>Koenuma *et al.*, 1989<br>Yee *et al.*, 1989*a*<br>Lambert *et al.*, 1990 |
| Hepatoma | Variable ± foetal pattern of IGF-II mRNA | 25k BP1 | Type I, II and insulin | IGF-I | IGF receptors increased in foetal, regenerating or malignant hepatocytes > normal. Hepatoma cells retain normal responses to GH and insulin | Heaton *et al.*, 1986<br>Cariani *et al.*, 1988<br>Caro *et al.*, 1988<br>Lee *et al.*, 1988<br>Hartshorn *et al.*, 1989<br>Su *et al.*, 1989<br>Singh *et al.*, 1990 |
| Pancreas | IGF-I | | Type I | IGF-I | | Ohmura *et al.*, 1990 |
| Renal adenocarcinoma | | 34k | Type I | | No consistent difference in BP production or IGF binding between normal and malignant tissue | Pekonen *et al.*, 1989 |
| Endometrial | | 37k, 40k (BP3)<br>32k (BP2) | Type I | | More binding sites on tumour than normal endometrium | Lamson *et al.*, 1989<br>Talavera *et al.*, 1990 |
| Neural/neuroendocrine meningioma, glioma | | | Type I | IGF-I, insulin | No IGF-I binding sites on normal leptomeninges. Insulin induces differentiation in cultured glioma and meningioma cells | Glick *et al.*, 1989<br>Kurihara *et al.*, 1989 |
| phaeochromocytoma | IGF-II | | | | IGF-II expression higher than normal adrenal medulla | Haselbacher *et al.*, 1987<br>Suzuki *et al.*, 1989 |
| Sarcoma { fibro- lipo- rhabdo leiomyo- } | IGF-II | | | | | De Larco & Todaro, 1978<br>Hume *et al.*, 1978<br>Scott *et al.*, 1985<br>Tricoli *et al.*, 1986<br>Gloudemans *et al.*, 1990 |
| { Ewings osteo- haemangio- } | IGF-I | | | IGF-I | | Blatt *et al.*, 1984<br>Pavelic *et al.*, 1985<br>Yee *et al.*, 1989*a*<br>Pollak & Richard, 1990 |
| Choriocarcinoma | IGF-II mRNA | | Type I | | | Ritvos *et al.*, 1988 |
| Embryonal carcinoma | High MW IGF-II | 35k | Type I, II | IGFs, insulin | Retinoic acid-induced differentiation stimulates synthesis of BP and high MW IGF-II | Heath & Shi, 1986<br>Biddle *et al.*, 1988<br>Weima *et al.*, 1989 |

MTC = medullary thyroid carcinoma. GH = growth hormone.

because of the relatively recent identification of the potential importance of IGFs in tumour biology, and also because of recognition that the widespread nature of IGFs and IGF receptors is likely to pose a problem in terms of treatment localisation. In general there have been two approaches to this problem: firstly to block IGF synthesis/secretion, and hence to reduce local or systemic IGF levels, and secondly to block the interaction of IGFs with their receptors.

In rats with chemically-induced mammary tumours, chronic calorie restriction is associated with lowered serum IGF-I and insulin levels and also with reduction in tumour incidence and multiplicity (Ruggieri *et al.*, 1989). Clinical studies have used endocrine treatments in an attempt to achieve the same effect. In normal postmenopausal women and men with prostate cancer, $E_2$ treatment is associated with a fall in circulating IGF-I levels. This is presumably a direct inhibitory effect on IGF-I production, given that it is accompanied by enhancement of growth hormone secretion (Dawson-Hughes *et al.*, 1986; Stege *et al.*, 1987). In postmenopausal patients, tamoxifen treatment is associated with a fall in growth hormone levels, presumably by blocking ERs at the hypothalamic-pituitary axis (Jordan, 1990). Serum IGF-I levels are lower in breast cancer patients on tamoxifen than in control patients of comparable age and status of disease and nutrition (Colletti *et al.*, 1989; Pollak *et al.*, 1990). It has been suggested that this could explain the known ability of tamoxifen to induce remissions in patients whose tumours are ER negative (Jordan, 1990).

The long-acting somatostatin analogue octreotide (SMS 201-995) has been shown to reduce circulating levels of various peptides including IGF-I in patients with acromegaly, carcinoid and other neuroendocrine tumours, and this is associated in some cases with measurable tumour regression (Kvols *et al.*, 1986; Lamberts *et al.*, 1987; Schally, 1988). Pollak *et al.* (1989) treated eight patients with non-endocrine solid tumours of exocrine pancreas, ovary, breast, kidney and colon. Octreotide therapy was accompanied by a significant fall in basal and arginine-stimulated growth hormone secretion and in serum IGF-I levels, but no data were given on tumour response. In 20 patients with SCLC, octreotide treatment was associated with a fall in circulating IGF-I levels in most patients, but there were no objective clinical responses (Macaulay *et al.*, 1991). At present it is not clear whether it will be possible to suppress circulating levels of a potent mitogen such as IGF-I to the extent that the local tissue concentration becomes limiting for tumour growth.

There seems more promise in attempts to inhibit the actions of IGFs by blocking their interaction with the receptor. A monoclonal antibody to IGF-I, SM1.2 (Russell *et al.*, 1984) has been shown to inhibit the growth of SCLC and non-SCLC cells *in vitro* (Minuto *et al.*, 1988; Macaulay *et al.*, 1990). However, this approach is unlikely to be successful *in vivo*, because of the substantial serum reservoir of IGF-I. In theory a membrane bound target is preferable, because there is unlikely to be a significant serum reservoir which would impair the localisation of treatment to the tumour. In many of the experimental systems described above, the mitogenic effects of IGFs and insulin appear to be mediated via the type I IGF receptor, and therefore this has been the treatment target chosen by most groups. A monoclonal antibody to the type I receptor, $\alpha IR_3$ (Kull *et al.*, 1983) has been shown to inhibit the growth of breast cancer cells *in vitro* (Rohlik *et al.*, 1987; Pollak *et al.*, 1988; Freed & Herington, 1989; Arteaga & Osborne, 1989; Cullen *et al.*, 1990). In serum-supplemented medium $\alpha IR_3$ inhibits the growth of ER positive and ER negative cell lines. In serum-free defined medium, $\alpha IR_3$ blocks the mitogenic effects of exogenous IGF-I and IGF-II, but does not inhibit basal or $E_2$-stimulated growth (Arteaga & Osborne, 1989; Cullen *et al.*, 1990). This is consistent with reports that breast cancer cells do not produce authentic IGF-I (Yee *et al.*, 1989*a*), and that the effects of $E_2$ in breast cancer cells are not mediated solely by IGF-I. *In vivo*, $\alpha IR_3$ causes dose-dependent inhibition of tumour take rate and growth of hormone-insensitive breast cancer xenografts in nude mice, but has no effect on the hormone-sensitive cell line MCF7 (Arteaga *et al.*, 1989). In addition, $\alpha IR_3$ has been shown to inhibit the growth *in vitro* of human SCLC (Nakanishi *et al.*, 1988; Macaulay *et al.*, 1990), pancreatic carcinoma (Ohmura *et al.*, 1990) and neuroblastoma (El-Badry *et al.*, 1989). It also inhibits growth of Wilms' tumour *in vitro* and *in vivo* (Gansler *et al.*, 1989). It may be that the use of this approach clinically will be confounded by the widespread nature of IGF receptors in normal tissues. However, antibody localisation may be favoured by the over-expression of IGF receptors by some tumours compared with the corresponding normal tissues.

There are two further ways of blocking the interaction of IGFs with receptors. The polyanionic compound suramin has been shown to inhibit the mitogenic effects of PDGF and EGF, and has recently been shown to interfere with the interaction between IGF-I and the type I receptor in cultured osteosarcoma cells (Pollak & Richard, 1990). This is associated with inhibition of IGF-I stimulated proliferation of these cells *in vitro*, an effect which is reversed by removal of the drug, suggesting a cytostatic rather than a cytotoxic effect. Finally, structure-activity studies indicate that distinct domains in the IGF-I molecule are responsible for type I receptor binding and mitogenic activiy (Cascieri *et al.*, 1988*b*, Chen *et al.*, 1988). Therefore it might be possible to synthesise an analogue of IGF-I which retains high affinity type I receptor binding, but which does not activate the receptor, thus producing an antagonistic effect on growth.

### Tumour hypoglycaemia

This rare metabolic manifestation of IGF activity has been described in patients with mesenchymal tumours including mesothelioma, fibrosarcoma, rhabdomyosarcoma, leiomyosarcoma, liposarcoma and haemangiopericytoma. It can also occur in cases of hepatoma, adrenocortical carcinoma, renal carcinoma, Wilms', and cancers of the breast, prostate and cervix. The tumours are often large and slow growing and two thirds are in the abdomen or retroperitoneum, the remainder being intrathoracic. Patients are characteristically elderly, and present with symptoms of hypoglycaemia, especially confusion, usually preceding the diagnosis of the tumour. Symptoms may be severe, requiring glucose infusion, and resolve after surgical resection of the tumour (Daughaday, 1989).

For some time it has been recognised that insulin is not the cause of hypoglycaemia complicating non-islet cell tumours. Recent molecular studies have confirmed earlier suggestions that this phenomenon is mediated by IGF production by the tumour. Northern analysis and RNase protection assay of three tumours (pleural mesothelioma, thoracic and pelvic fibrosarcomas) revealed elevated levels of IGF-II mRNA. There were high circulating levels of immunoreactive IGF-II, most of which was in a high MW (9,000–15,000) form (Daughaday *et al.*, 1988; Ron *et al.*, 1989; Daughaday, 1990). Other studies have confirmed over-expression of IGF-II mRNA, without IGF-II gene amplification or rearrangement (Lowe *et al.*, 1989). It is suggested that IGF-II causes hypoglycaemia by stimulating glucose uptake into peripheral tissues (fat and muscle) and possibly also into the tumour. In addition, a negative feed-back effect on growth hormone secretion impairs the hepatic response to hypoglycaemia, and suppresses serum IGF-I levels (Axelrod & Ron, 1988). However, several groups have been unable to show any elevation of circulating IGF-II in patients with tumour hypoglycaemia (Widmer *et al.*, 1983; Merimee, 1986). One patient with a para-ovarian sarcoma, hypoglycaemia and high tumour IGF-II mRNA had suppressed serum IGF-I and IGF-II, presumably as a consequence of the hypoglycaemia, and neither the tumour cyst fluid nor the primary tissue culture conditioned medium had high IGF-II levels (Schofield *et al.*, 1989). This over-expression of mRNA without apparent increase in peptide production is analogous to the situation described in Wilms' (Haselbacher *et al.*, 1987).

Two aspects of IGF biology may explain the apparent lack of correlation between the degree of hypoglycaemia and measured levels of circulating IGF-II. Firstly, several studies have demonstrated the presence of high MW incompletely processed pro-IGF-II in tumours and sera of hypoglycaemic patients with hepatoma and fibrosarcoma but not in euglycaemic patients with hepatoma (Daughaday *et al.*, 1988 and 1990; Shapiro *et al.*, 1990). This 'big' IGF-II is fully reactive with the IGF-II receptor (Daughaday *et al.*, 1988) but has lower affinity for serum IGF BPs than authentic IGF-II (Shapiro *et al.*, 1990). Secondly, the 150,000 MW IGF BP complex appears to be absent from the serum of some patients with tumour hypoglycaemia, replaced in some cases with 110,000 and 60,000 MW complexes (Daughaday & Kapachia, 1989). Thus it is possible that a greater proportion of circulating IGF-II is unbound, or that the reduction in IGF-II/BP binding affinity is sufficient to alter the equilibrium in favour of IGF-II binding to cell surface receptors. Also, the IGF-II in lower MW complexes may penetrate better into tissues, again facilitating interaction with the receptor. These factors may explain the severe symptoms of hypoglycaemia which can occur in the presence of modest no elevation in absolute levels of serum IGF-II.

### Conclusions

This review has covered the two main manifestations of inappropriate or excessive IGF activity in cancer. Tumour hypoglycaemia is a rare paraneoplastic manifestation of the metabolic effects of IGFs. Of potentially greater importance is the role of IGFs as tumour growth factors. IGF-I has been implicated in growth regulation of a variety of neoplasia especially the common solid tumours, and IGF-II in embryonal tumours. This parallels the importance of IGF-I in normal post-natal growth, and the suggestion that IGF-II is an important regulator of foetal development. Notably, exclusive paternal expression of IGF-II has been linked with a foetal overgrowth syndrome, BWS, and with the embryonal tumours associated with it. Recent studies have questioned whether tumours are capable of synthesising authentic IGF-I, but there is little doubt that many neoplastic cells express IGF receptors and show growth enhancement in response to IGFs derived from serum or released locally by stromal cells. Although the same is true of many normal tissues, the increased IGF binding capacity of tumour tissue compared with its normal counterpart may favour localisation to the tumour of any IGF-directed treatment. The value of this approach is currently being tested experimentally, but has yet to be evaluated clinically.

I am grateful to Professor Judson Van Wyk and Dr Jeff Holly for their advice, and to Mrs Julia Holborn for typing the manuscript.

### References

ARTEAGA, C.L. & OSBORNE, C.K. (1989). Growth inhibition of human breast cancer cells *in vitro* with an antibody against the type I somatomedin receptor. *Cancer Res.*, **49**, 6237.

ARTEGA, C.L., KITTEN, L.J., CORONADO, E.B., JACOB, S., KULL, F.C. Jr, ALLRED, D.C. & OSBORNE, C.K. (1989). Blockade of the type I somatomedin receptor inhibits growth of human breast cancer cells in athymic mice. *J. Clin. Invest.*, **64**, 1418.

AXELROD, L. & RON, D. (1988). Insulin-like growth factor II and the riddle of tumor-induced hypoglycemia. *N. Engl. J. Med.*, **319**, 1477.

BAGLEY, C.J., MAY, B.L., SZABO, L. & 5 others (1989). A key functional role for the insulin-like growth factor I N-terminal pentapeptide. *Biochem. J.*, **259**, 665.

BARRECA, A. & MINUTO, F. (1989). Somatomedins: chemical and functional characteristics of the different molecular forms. *J. Endocrinol. Invest.*, **12**, 279.

BAXTER, R.C. (1988). Characterisation of the acid-labile subunit of the growth hormone-dependent insulin-like growth factor binding protein complex. *J. Clin. Endocrinol. Metabol.*, **67**, 265.

BECKWITH, J.B. (1963). Extreme cytomegaly of the adrenal fetal cortex, omphalocele, hyperplasia of kidneys and pancreas, and leydig-cell hyperplasia: another syndrome? Western Society for Pediatric Research, Los Angeles, California.

BIDDLE, C., LI, C.H., SCHOFIELD, P.N. & 5 others (1988). Insulin-like growth factors and the multiplication of tera-2, a human teratoma-derived cell line. *J. Cell Sci.*, **90**, 475.

BLATT, J., WHITE, C., DIENES, S., FRIEDMAN, H. & FOLEY, T.P. Jr. (1984). Production of insulin-like growth factor by osteosarcoma. *Biochem. Biophys. Res. Commun.*, **123**, 373.

BRICE, A.L., CHEETHAM, J.E., BOLTON, V.N., HILL, N.C. & SCHOFIELD, P.N. (1989). Temporal changes in the expression of the insulin-like growth factor II gene associated with tissue maturation in the human fetus. *Development*, **106**, 543.

CARIANI, E., LASSERRE, C., SERUIN, D. & 6 others (1988). Differential expression of insulin-like growth factor II mRNA in human primary liver cancers, benign liver tumors and liver cirrhosis. *Cancer Res.*, **48**, 6844.

CARO, J.F., POULOS, J., ITTOOP, O., PORIES, W.J., FLICKINGER, E.G. & SINHA, M.K. (1988). Insulin-like growth factor I binding in hepatocytes from human liver, human hepatoma and normal, regenerating and fetal rat liver. *J. Clin. Invest.*, **81**, 976.

CASCIERI, M.A., SAPERSTEIN, R., HAYES, N.S. & 4 others (1988*a*). Serum half-life and biological activity of mutants of human insulin-like growth factor I which do not bind to serum binding proteins. *Endocrinol.*, **123**, 373.

CASCIERI, M.A., CHICCHI, G.G., APPLEBAUM, J., HAYES, N.S., GREEN, B.G. & BAYNE, M.L. (1988*b*). Mutants of human insulin-like growth factor I with reduced affinity for the type I insulin-like growth factor receptor. *Biochem.*, **27**, 3229.

CAVAILLES, V., GARCIA, M. & ROCHEFORT, H. (1989). Regulation of cathepsin-D and pS2 gene expression by growth factors in MCF7 human breast cancer cells. *Mol. Endocrinol.*, **3**, 552.

CHEN, Z.Z., SCHWARTZ, G.P., ZONG, L., THOMPSON BURKE, G.T., CHANLEY, J.D. & KATSOYANNIS, P.G. (1988). Determinants of growth-promoting activity reside in the A-domain of insulin-like growth factor I. *Biochem.*, **27**, 6105.

CLEMMONS, D.R. & VAN WYK, J.J. (1981). Somatomedin-C and platelet-derived growth factor stimulate human fibroblast replication. *J. Cell Physiol.*, **106**, 361.

COLLETTI, R.B., ROBERTS, J.D., DEVLIN, J.T. & COPELAND, K.C. (1989). Effect of tamoxifen on plasma insulin-like growth factor I in patients with breast cancer. *Cancer Res.*, **49**, 1882.

CULLEN, K.J., YEE, D., SLY, W.S. & 4 others (1990). Insulin-like growth factor receptor expression and function in human breast cancer. *Cancer Res.*, **50**, 48.

CZECH, M.P. (1989). Signal transmission by the insulin-like growth factors. *Cell*, **59**, 235.

DAUGHADAY, W.H., HALL, K., SALMON, W.D., VAN DEN BRANDE, J.L. & VAN WYK, J.J. (1987). Letter to the Editor: on the nomenclature of the somatomedins and insulin-like growth factors. *J. Clin. Endocrinol. Metabol.*, **65**, 1075.

DAUGHADAY, W.H., EMANUELLE, M.A., BROOKS, M.H., BARBATO, A.L., KAPADIA, M. & ROTWEIN, P. (1988). Synthesis and secretion of insulin-like growth factor II by a leiomyosarcoma with associated hypoglycaemia. *N. Engl. J. Med.*, **319**, 1434.

DAUGHADAY, W.H. & ROTWEIN, P. (1989). Insulin-like growth factors I and II. Peptide, messenger ribonucleic acid and gene structures, serum, and tissue concentrations. *Endocrine Rev.*, **10**, 68.

DAUGHADAY, W.H. & KAPADIA, M. (1989). Significance of abnormal serum binding of insulin-like growth factor II in the development of hypoglyemia in patients with non-islet-cell tumors. *Proc. Natl Acad. Sci. USA*, **86**, 6778.

DAUGHADAY, W.H. (1989). Hypoglycemia in patients with non-Islet cell tumors. *Endocrinol. Metabol. Clin. N. Am.*, **18**, 91.

DAUGHADAY, W.H. (1990). Editorial: the possible autocrine paracrine and endocrine roles of insulin-like growth factors of human tumors. *Endocrinol.*, **127**, 1.

DAUGHADAY, W.H., WU, J.-C., LEE, S.-D. & KAPADIA, M. (1990). Abnormal processing of pro-IGF-II in patients with hepatoma and in some hepatitis B virus antibody-positive asymptomatic individuals. *J. Lab. Clin. Med.*, **116**, 555.

DAVENPORT, M.L., D'ERCOLE, A.J., AZIZKHAN, J.C. & LUND, P.K. (1988). Somatomedin-C insulin-like growth factor and insulin-like growth factor II mRNAs during lung development in the rat. *Exp. Lung Res.*, **14**, 607.

DAWSON-HUGHS, B., STERN, D., GOLDMAN, J. & REICHLIN, S. (1986). Regulation of growth hormone and somatomedin-C secretion in postmenopausal women: effect on physiological estrogen replacement. *J. Clin. Endocrinol. Metab.*, **63**, 424.

DE LARCO, J.E. & TODARO, G.J. (1978). A human fibrosarcoma cell line producing multiplication stimulating activity (MSA)-related peptides. *Nature*, **272**, 356.

DE LEON, D.D., WILSON, D.M., BAKKER, B., LAMSOM, G., HINTZ, R.L. & ROSENFELD, R.G. (1989). Characterisation of insulin-like growth factor binding proteins from human breast cancer cells. *Mol. Endocrinol.*, **3**, 567.

DECHIARA, T.M., EFSTRATIADIS, A. & ROBERTSON, E.J. (1990). A growth-deficiency phenotype in heterozygous mice carrying an insulin growth factor II gene disrupted by targetting. *Nature*, **345**, 78.

D'ERCOLE, J.A., STILES, A.D. & UNDERWOOD, L.E. (1984). Tissue concentrations of somatomedin C: further evidence for multiple sites of synthesis and paracrine or autocrine mechanisms of action. *Proc. Natl Acad. Sci. USA*, **81**, 935.

DICKSON, R.B., MCMANAWAY, M. & LIPPMAN, M.E. (1986). Estrogen induced factors of breast cancer cells partially replace estrogen to promote tumour growth. *Science*, **213**, 1540.

DICKSON, R.B., KASID, A., HUFF, K.K. & 5 others (1987). Activation of growth factor secretion in tumorigenic states of breast cancer induced by 17 beta-estradiol or v-Ha-*ras* oncogene. *Proc. Natl Acad. Sci. USA*, **84**, 837.

DULAK, N. & TEMIN, H.N. (1973). Partially purified polypeptide fraction from rat liver cell conditioned medium with multiplication-stimulating activity for embryo fibroblasts. *J. Cell. Physiol.*, **81**, 153.

EL-BADRY, O.M., ROMANUS, J.A., HELMAN, L.J., COOPER, M.J., RECHLER, M.M. & ISRAEL, M.A. (1989). Autonomous growth of a human neuroblastoma cell line is mediated by insulin-like growth factor II. *J. Clin. Invest.*, **84**, 829.

EL-BADRY, O.M., HELMAN, L.J., CHATTEN, J., STEINBERG, S.M., EVANS, A.E. & ISRAEL, M.A. (1991). Insulin-like growth factor II-mediated proliferation of human neuroblastoma. *J. Clin. Invest.*, **87**, 648.

FAGIN, J.A. & MELMED, S. (1987). Relative increase in insulin-like growth factor I messenger ribonucleic acid levels in compensatory renal hypertrophy. *Endocrinol.*, **120**, 718.

FOEKENS, J.A., PORTENGEN, H., JANSSEN, M. & KLIJN, J.G.M. (1989*a*). Insulin-like growth factor-1 receptors and insulin-like growth factor-1-like activity in human primary breast cancer. *Cancer*, **63**, 2139.

FOEKENS, J.A., PROTENGEN, H., VAN PUTTEN, W.L. & 4 others (1989*b*). Prognostic value of receptors for insulin-like growth factor I, somatostatin, and epidermal growth factor in human breast cancer. *Cancer Res.*, **49**, 7002.

FREED, K.A. & HERINGTON, A.C. (1989). Insulin-like growth factor-I and its autocrine role in growth of MCF-7 human breast cancer cells in culture. *J. Mol. Endocrinol.*, **3**, 183.

FROESCH, E.R., BURGI, H., RAMSEIER, E.B., BALLY, P. & LABHART, A. (1963). Antibody suppressible and non-suppressible insulin-like activities in human serum and their physiologic significance. *J. Clin. Invest.*, **42**, 1816.

FROESCH, E.R. & ZAPF, J. (1985). Insulin-like growth factors and insulin: comparative aspects. *Diabetologia*, **28**, 485.

FURLANETTO, R.W. & DICARLO, J.N. (1984). Somatomedin-C receptors and growth effects in human breast cells maintained in long-term tissue culture. *Cancer Res.*, **44**, 2122.

GANSLER, T., FURLANETTO, R., STOKES GRAMLING, T. & 5 others (1989). Antibody to type I insulin-like growth factor receptor inhibits growth of Wilms' tumour in culture and in athymic mice. *Am. J. Pathol.*, **135**, 961.

GLICK, R.P., GETTLEMAN, R., PATEL, K., LAKSHMAN, R. & TSIBRIS, J.C.M. (1989). Insulin and insulin-like growth factor I in brain tumors: binding and *in vitro* effects. *Neurosurg.*, **24**, 791.

GLOUDEMANS, T., PRINSEN, I., VAN UNNIK, J.A.M., LIPS, C.J.M., DEN OTTER, W. & SUSSENBACK, J.S. (1990). Insulin-like growth factor gene expression in human smooth muscle tumours. *Cancer Res.*, **50**, 6689.

GOLDSTEIN, R.H., POLIKS, C.F., PILCH, P.F., SMITH, B.D. & FINE, A. (1989). Stimulation of collagen formation by insulin and insulin-like growth factor I in cultures of human lung fibroblasts. *Endocrinol.*, **124**, 964.

GULER, H.-P., ZAPF, J., SCHMID, C. & FROESCH, E.R. (1987). Short-term metabolic effects of recombinant human insulin-like growth factor I in healthy adults. *N. Engl. J. Med.*, **317**, 137.

GULER, H.-P., ZAPF, J., SCHMID, C. & FROESCH, E.R. (1989). Insulin-like growth factors I and II in healthy man. Estimations of half-lives and production rates. *Acta Endocrinol.*, **121**, 753.

HAN, V.K.M., D'ERCOLE, A.J. & LUND, P.K. (1987). Cellular localisation of somatomedin (insulin-like growth factor) messenger RNA in the human fetus. *Science*, **236**, 193.

HARTSHORN, M.A., SCOTT, C.D. & BAXTER, R.C. (1989). Immunofluorescent localisation of type II insulin-like growth factor receptor in rat liver and hepatoma cells. *J. Endocrinol.*, **121**, 221.

HASELBACHER, G.K., IRMINGER, J.-C., ZAPF, J., ZIEGLER, W.H. & HUMBEL, R.E. (1987). Insulin-like growth factor II in human adrenal pheochromocytomas and Wilms' tumors: expression at the mRNA and protein level. *Proc. Natl Acad. Sci. USA*, **84**, 1104.

HEATH, J.K. & SHI, W.-K. (1986). Developmentally regulated expression of insulin-like growth factors by differentiated murine teratocarcinomas and extraembryonic mesoderm. *J. Embryol. Exp. Morph.*, **95**, 193.

HEATON, J.H., KRETT, N.L. & GELEHRTER, T.D. (1986). Regulation of insulin and insulin-like growth factor (IGF) responsiveness by IGFs in rat hepatoma cells. *Endocrinol.*, **118**, 2555.

HENRY, I., BONAITI-PELLIE, C., CHEHENSSE, V. & 4 others (1991). Uniparental paternal disomy in a genetic cancer-predisposing syndrome. *Nature*, **351**, 665.

HOLLY, J.M.P. & WASS, J.A.H. (1989). Insulin-like growth factors: autocrine, paracrine or endocrine? New perspectives of the somatomedin hypothesis in the light of recent developments. *J. Endocrinol.*, **122**, 611.

HOSSENLOPP, P., SEURIN, D., SEGOVIA-QUINSON, B., HARDOUIN, S. & BINOUX, M. (1986). Analysis of serum insulin-like growth factor binding proteins using Western blotting: use of the method of titration of the binding proteins and competitive binding studies. *Anal. Biochem.*, **154**, 138.

HUFF, K.K., KAUFMAN, D., GABBAY, K.H., SPENCER, E.M., LIPPMAN, M.E. & DICKSON, R.B. (1986). Secretion of an insulin-like growth factor-I-related protein by human breast cancer cells. *Cancer Res.*, **46**, 4613.

HUFF, K.K., KNABBE, C., LINDSEY, R. & 4 others (1988). Multihormonal regulation of insulin-like growth factor-I-related protein in MCF-7 human breast cancer cells. *Molec. Endocrinol.*, **2**, 200.

HUMBEL, R.E. (1990). Review. Insulin-like growth factors I and II. *Eur. J. Biochem.*, **190**, 445.

HUME, D.A., HANSEN, K., WEIDEMANN, M.J. & FERBER, E. (1978). A human fibrosarcoma cell line producing multiplication stimulating activity-related peptides. *Nature*, **272**, 356.

IRMINGER, J.C., SCHOENLE, E.J., BRINER, J. & HUMBEL, R.E. (1989). Structural alteration of the insulin-like growth factor II-gene in Wilms' tumour. *Eur. J. Pediatr.*, **148**, 620.

JAQUES, G., ROTSCH, M., WEGMANN, C., WORSCH, U., MAASBERG, M. & HAVEMANN, K. (1988). Production of immunoreactive insulin-like growth factor I and response to exogenous IGF-I in small cell lung cancer cell lines. *Exp. Cell Res.*, **176**, 336.

JAQUES, G., KIEFER, P., ROTSCH, M. & 4 others (1989). Production of insulin-like growth factor binding proteins by small cell lung cancer cell lines. *Exp. Cell Res.*, **184**, 396.

JORDAN, V.C. (1990). Editorial. Estrogen receptor-mediated direct and indirect antitumour effects of tamoxifen. *J. Natl Cancer Inst.*, **82**, 1662.

KALEKO, M., RUTTER, W.J. & MILLER, A.D. (1990). Overexpression of the human insulin-like growth factor I receptor promotes ligand-dependent neoplastic transformation. *Mol. Cell. Biol.*, **10**, 464.

KATZENELLENBOGEN, B.S. & NORMAN, M.J. (1990). Multihormonal regulation of the progesterone receptor in MCF-7 human breast cancer cells: interrelationships among insulin insulin-like growth factor-I, serum, and estrogen. *Endocrinol.*, **126**, 891.

KLAPPER, D.G., SVOBODA, M.E. & VAN WYK, J.J. (1983). Sequence analysis of somatomedin-C: confirmation of identity with insulin-like growth factor I. *Endocrinol.*, **112**, 2215.

KOENUMA, M., YAMORI, T. & TSURO, T. (1989). Insulin and insulin-like growth factor I stimulate proliferation of metastatic variants of colon carcinoma 26. *Jpn J. Cancer Res.*, **80**, 51.

KOHN, E.C., FRANCIS, E.A., LIOTTA, L.A. & SCHIFFMANN, E. (1990). Heterogeneity of the motility responses in malignant tumor cells: a biological basis for the diversity and homing of metastatic cells. *Int. J. Cancer*, **46**, 287.

KULL, F.C.S., JACOBS, S., SU, Y.-F., SVOBODA, M.E., VAN WYK, J.J. & CUATRECASAS, P. (1983). Monoclonal antibodies to receptor for insulin and somatomedin-C. *J. Biol. Chem.*, **258**, 6561.

KURIHARA, M., TOKUNAGA, Y., TSUTSUMI, K. & 4 others (1989). Characterisation of insulin-like growth factor I and epidermal growth factor receptors in meningioma. *J. Neurosurg.*, **71**, 538.

KVOLS, L.K., MORTEL, C.G., O'CONNELL, M.J. & 3 others (1986). Treatment of the malignant carcinoid syndrome. Evaluation of a long-acting somatostatin analogue. *N. Engl. J. Med.*, **315**, 663.

LABURTHE, M., ROUYER-FESSARD, C. & GAMMELTOFT, S. (1988). Receptors for insulin-like growth factors I and II in rat gastrointestinal epithelium. *Am. J. Physiol.*, **254**, G457.

LAMBERT, S., VIVARIO, J., BONIVER, J. & GOL-WINKLER, R. (1990). Abnormal expression and structural modification of the insulin-like growth factor-II gene in human colorectal tumors. *Int. J. Cancer*, **46**, 405.

LAMBERTS, S.W.J. & UITTERLINDEN DEL POZO, E. (1987). SMS 201-995 induces a continuous decline in circulating growth hormone and somatomedin-C levels during therapy of acromegalic patients for over two years. *J. Clin. Endocrinol. Metabol.*, **65**, 703.

LAMMERS, R., GRAY, A., SCHLESSINGER, J. & ULLRICH, A. (1989). Differential signalling potential of insulin- and IGF-I receptor cytoplasmic domains. *EMBO J.*, **8**, 1369.

LAMSON, G., OH, Y., PHAM, H., GIUDICE, L.C. & ROSENFELD, R.G. (1989). Expression of two insulin-like growth factor binding proteins in a human endometrial cancer cell line: structural, immunological and genetic characterisation. *J. Clin. Endocrinol. Metabol.*, **69**, 852.

LEE, Y.-L., HINTZ, R.L., JAMES, P.M., LEE, P.D.K., SHIVELY, J.E. & POWELL, D.R. (1988). Insulin-like growth factor (IGF) binding protein complementary deoxyribonucleic acid from human HEP G2 hepatoma cells: predicted protein sequence suggests an IGF binding domain different from those of the IGF-I and IGF-II receptors. *Mol. Endocrinol.*, **2**, 404.

LITTLE, M.H., ABLETT, G. & SMITH, P.J. (1987). Enhanced expression of insulin-like growth factor II is not a necessary event in Wilms' tumour progression. *Carcinogenesis*, **8**, 865.

LITTLE, M., VAN HEYNINGEN, V. & HASTIE, N. (1991). Dads and disomy and disease. *Nature*, **351**, 609.

LOWE, W.L., ROBERTS, C.T., LEROITH, D. & 10 others (1989). Insulin-like growth factor-II in non-islet cell tumours associated with hypoglycemia: increased levels of messenger ribonucleic acid. *J. Clin. Endocrinol. Metabol.*, **69**, 1153.

MACAULAY, V.M., TEALE, J., EVERARD, M.J., JOSHI, G.P., SMITH, I.E. & MILLAR, J.L. (1988*a*). Somatomedin-C insulin-like growth factor I is a mitogen for human small cell lung cancer. *Br. J. Cancer*, **57**, 91.

MACAULAY, V.M., TEALE, J.D., EVERARD, M.J., JOSHI, G.P., MILLAR, J.L. & SMITH, I.E. (1988*b*). Serum insulin-like growth factor-I levels in patients with small cell lung cancer. *Eur. J. Cancer Clin. Oncol.*, **24**, 1241.

MACAULAY, V.M., EVERARD, M.J., TEALE, J.D. & 4 others (1988*b*). Autocrine function for insulin-like growth factor I in human small cell lung cancer cell lines and fresh tumor cells. *Cancer Res.*, **50**, 2511.

MACAULAY, V.M., SMITH, I.E., EVERARD, M.J., TEALE, J.D., REUBI, J.-C. & MILLAR, J.L. (1991). Experimental and clinical studies with somatomedin analogue octreotide in small cell lung cancer. *Br. J. Cancer*, (in press).

MACDONALD, R.G., PFEFFER, S.R., COUSSENS, L. & 7 others (1988). A single receptor binds both insulin-like growth factor II and mannose-6-phosphate. *Science*, **239**, 1134.

MARQUARDT, H., TODARO, G.J., HENDERSON, L.E. & OROSZLAN, S. (1981). Purification and primary structure of polypeptide with multiplicating-stimulating activity from rat liver cell cultures. *J. Biol. Chem.*, **256**, 6859.

MASSAGUE, J. & CZECH, M.P. (1982). The subunit structures of two distinct receptors for insulin-like growth factors I and II and their relationship to the insulin receptor. *J. Biol. Chem.*, **257**, 5038.

MATHEWS, L.S., HAMMER, R.E., BEHRINGER, R.R. & 4 others (1988). Growth enhancement of transgenic mice expressing human insulin-like growth factor I. *Endocrinol.*, **123**, 2827.

MERIMEE, T.J. (1986). IGFs in patients with non-islet cell tumours and hypoglycaemia. *Metabolism*, **35**, 360.

MINUTO, F., DEL MONTE, P., BARRECA, A. & 4 others (1986). Evidence for an increased somatomedin C insulin-like growth factor I content in primary hung lung tumors. *Cancer Res.*, **46**, 985.

MINUTO, F., DEL MONTE, P., BARRECA, A., ALAMA, A., CARIOLA, G. & GIORDANO, G. (1988). Evidence for autocrine mitogenic stimulation by somatomedin-C insulin-like growth factor I on an established human lung cancer cell line. *Cancer Res.*, **48**, 3716.

MINUTO, F., BARRECA, A., DEL MONTE, P., CARIOLA, G., TORRE, G.C. & GIORDANO, G. (1989). Immunoreactive insulin-like growth factor I (IGF-I) and IGF-I binding protein content in human thyroid tissue. *J. Clin. Endocrinol. Metabol.*, **68**, 621.

MORGAN, D.O., EDMAN, J.C., STANDRING, D.N. & 4 others (1987). Insulin-like growth factor II receptor as a multifunctional binding protein. *Nature*, **329**, 301.

MURPHY, L.J. & FRIESEN, H.G. (1988). Differential effects of estrogen and growth hormone on uterine and hepatic insulin-like growth factor I gene expression in the ovariectomised hypophysectomised rat. *Endocrinol.*, **122**, 1011.

NAKANISHI, Y., MULSHINE, J.L., KASPRZYK, P.G. & 7 others (1988). Insulin-like growth factor-I can mediate autocrine proliferation of human small cell lung cancer cell lines *in vitro*. *J. Clin. Investig.*, **82**, 354.

OHMURA, E., OKADA, M., ONODA, N. & 4 others (1990). Insulin-like growth factor I and transforming growth factors as autocrine growth factors in human pancreatic cancer cell growth. *Cancer Res.*, **50**, 103.

OKIMURA, Y., KITAJIMA, N., UCHIYAMA, T. & 4 others (1989). Insulin-like growth factor-I production and the presence of IGF-I receptors in rat medullary thyroid carcinoma cell line 6-23(clone 6). *Biochem. Biophys. Res. Commun.*, **161**, 589.

OSBORNE, K.C. & ARTEAGA, C.L. (1990). Autocrine and paracrine growth regulation of breast cancer: clinical implications. *Breast Cancer Res. Treat.*, **15**, 3.

OSHIMA, A., NOLAN, C.M., KYLE, J.W., GRUBB, J.H. & SLY, W.S. (1988). The human cation-independent mannose 6-phosphate receptor. Cloning and sequence of the full-length cDNA and expression of functional receptor in cos cells. *J. Biol. Chem.*, **263**, 2553.

PAVELIC, K., VRBANEC, D., MARUSIC, S., LEVANAT, S. & CABRIJAN, T. (1985). Autocrine tumor growth regulation by somatomedin C: an *in vitro* model. *J. Endocrinol.*, **109**, 233.

PEKONEN, F., PARTANEN, S., MAKINEN, T. & RUTANEN, E.-M. (1988). Receptors for epidermal growth factor and insulin-like growth factor I and their relation to steroid receptors in human breast cancer. *Cancer Res.*, **48**, 1343.

PEKONEN, F., PARTANEN, S. & RUTANEN, E.-M. (1989). Binding of epidermal growth factor and insulin-like growth factor I in renal carcinoma and adjacent normal kidney tissues. *Int. J. Cancer*, **43**, 1029.

PEYRAT, J.-P., BONNETERRE, J., BEUSCART, R., DIJANE, J. & DEMAILLE, A. (1988). Insulin-like growth factor I receptors in human breast cancer and their relation to estradiol and progesterone receptors. *Cancer Res.*, **48**, 6429.

PIERSON, R.W. Jr. & TEMIN, H.M. (1972). The partial purification from calf serum of a fraction with multiplication stimulating activity for chicken fibroblasts in cell culture and with non-suppressible insulin-like activity. *J. Cell Physiol.*, **79**, 319.

POLLAK, M.N., PERDUE, J.F., MARGOLESE, R.G., BAER, K. & RICHARD, M. (1987). Presence of somatomedin receptor on primary human breast and colon carcinomas. *Cancer Lett.*, **38**, 223.

POLLAK, M.N., POLYCHRONAKOS, C., YOUSEFI, S. & RICHARD, M. (1988). Characterisation of insulin-like growth factors I (IGF-I) receptors of human breast cancer cells. *Biomed. Biophys. Res. Comm.*, **154**, 326.

POLLAK, M.N., POLYCHRONAKOS, C. & GUYDA, H. (1989). Somatomedin analogue SMS 201-995 reduces serum IGF-I levels in patients with neoplasms potentially dependent on IGF-I. *Anticancer Res.*, **9**, 889.

POLLAK, M., COSTANTINO, J., POLYCHRONAKOS, C. & 5 others (1990). Effect of tamoxifen on serum insulin-like growth factor I levels in stage I breast cancer patients. *J. Natl Cancer Inst.*, **82**, 1693.

POLLAK, M. & RICHARD, M. (1990). Suramin blockade in insulin-like growth factor I stimulated proliferation of human osteosarcoma cells. *J. Natl Cancer Inst.*, **82**, 1349.

RECHLER, M.M. & NISSLEY, S.P. (1986). Insulin-like growth factor somatomedin receptor subtypes: structure, function, and relationships to insulin receptors and IGF carrier proteins. *Hormone Res.*, **24**, 152.

REEVE, A.E., ECCLES, M.R., WILKINS, R.J., BELL, G.I. & MILLOW, L.J. (1985). Expression of insulin-like growth factor-II transcripts in Wilms' tumour. *Nature*, **317**, 258.

REEVE, J.G., PAYNE, J.A. & BLEEHEN, N.M. (1990). Production of immunoreactive insulin-like growth factor-I (IGF-I) and IGF-I binding proteins by human lung tumours. *Br. J. Cancer*, **61**, 727.

RINDERKNECHT, E. & HUMBEL, R.E. (1978). Primary structure of human insulin-like growth factor II. *Febs Lett.*, **89**, 283.

RITVOS, O., RANTA, T., JALKANEN, J. & 4 others (1988). Insulin-like growth factor binding protein from human decidua inhibits the binding and biological action of IGF-I in cultured choriocarcinoma cells. *Endocrinol.*, **122**, 2150.

ROCHEFORT, H., CAPONY, F., GARCIA, M. & 5 others (1987). Estrogen-induced lysosomal proteases secreted by breast cancer cells: a role in carcinogenesis? *J. Cell Biochem.*, **35**, 17.

ROHLIK, Q.T., ADAMS, D., KULL, F.C. & JACOBS, S. (1987). An antibody to the receptor for insulin-like growth factor I inhibits the growth of MCF-7 cells in tissue culture. *Biomed. Biophys. Res. Comm.*, **149**, 276.

ROM, W.N., BASSET, P., FELLS, G.A., NUKIWA, T., TRAPNELL, B.C. & CRYSTAL, R.G. (1988). Alveolar macrophages release an insulin-like growth factor I type molecule. *J. Clin. Invest.*, **82**, 1685.

RON, D., POWERS, A.C., PANDIAN, M.R., GODINE, J.E. & AXELROD, L. (1989). Increased insulin-like growth factor II production and consequent suppression of growth hormone secretion: a dual mechanism for tumor-induced hypoglycemia. *J. Clin. Endocrinol. Metabol.*, **68**, 701.

ROSS, M., FRANCIS, G.L., SZABO, L., WALLACE, J.C. & BALLARD, F.J. (1989). Insulin-like growth factor binding protein inhibits the biological activities of IGF-I and IGF-2 but not des-(1,3)-IGF-1. *Biochem. J.*, **258**, 267.

ROTH, R.A. (1988). Structure of the receptor for insulin-like growth factor II: the puzzle amplified. *Science*, **239**, 1269.

ROTWEIN, P.T. (1986). Two insulin-like growth factor I messenger RNAs are expressed by human liver. *Proc. Natl. Acad. Sci. USA*, **83**, 177.

RUGGERI, B.A., KLURFELD, D.M., KRITCHEVSKY, D. & FURLANETTO, R.W. (1989). Caloric restriction and 7,12-Dimethylbenz(*a*) anthracene-induced mammary tumor growth in rats: alterations in circulating insulin, insulin-like growth factors I and II and epidermal growth factor. *Cancer Res.*, **49**, 4130.

RUSSELL, W.E., VAN WYK, J.J. & PLEDGER, W.J. (1984). Inhibition of the mitogenic effects of plasma by a monoclonal antibody to somatomedin C. *Proc. Natl Acad. Sci. USA*, **81**, 2389.

RUTANEN, E.M., PEKONEN, F. & MAKINEN, T. (1988). Soluble 23K binding protein inhibits the binding of insulin-like growth factor to its cell receptors in human secretory phase endometrium: evidence for autocrine paracrine regulation of growth factor action. *J. Clin. Endocrinol. Metabol.*, **66**, 173.

SALMON, W.D. Jr & DAUGHADAY, W.H. (1957). A hormonally controlled serum factor which stimulates sulfate incorporation by cartilage *in vitro*. *J. Lab. Clin. Med.*, **49**, 825.

SCHALLY, A.V. (1988). Oncological applications of somatostatin analogues. *Cancer Res.*, **48**, 6977.

SCHOENLE, E., ZAPF, J. & FROESCH, E.R. (1982). Insulin-like growth factor I and II stimulate growth of hypophysectomized rats. *Diabetologia*, **23**, 199.

SCHOENLE, E., ZAPF, J. & FROESCH, E.R. (1983). Long-term *in vivo* effects of insulin-like growth factors I and II on growth indices: direct evidence in favor of the somatomedin-hypothesis. In De Gruyter, W. (ed.) *Insulin-like Growth Factor Somatomedins: Basic Chemistry, Biology, Clinical Importance*. Spencer, E.M. Berlin NY, p. 51.

SCHOFIELD, P.N., CONNOR, H., TURNER, R.C. & ZAPF, J. (1989). Tumour hypoglycaemia: raised tumour IGF-II mRNA associated with reduced plasma somatomedins. *Br. J. Cancer*, **60**, 661.

SCOTT, J., COWELL, J., ROBERTSON, M.E. & 8 others (1985). Insulin-like growth factor II gene expression in Wilms' tumour and embryonic tissues. *Nature*, **317**, 260.

SHAPIRO, E.T., BELL, G.I., POLONSKY, K.S., RUBENSTEIN, A.H., KEW, M.C. & TAGER, H.S. (1990). Tumor hypoglycemia: relationship to high molecular weight insulin-like growth factor-II. *J. Clin. Invest.*, **85**, 1672.

SHIGEMATSU, K., KATAOKA, Y., KAMIO, T., KARIHARA, M, NIWA, M. & TSUCHIYAMA, H. (1990). Partial characterisation of insulin-like growth factor I in primary human lung cancers using immunohistochemical and receptor autoradiographic techniques. *Cancer Res.*, **50**, 2481.

SIEGFRIED, J.M. (1989). Culture of primary lung tumors using medium conditioned by a lung carcinoma cell line. *J. Cell. Biochem.*, **41**, 91.

SINGH, A., HAMILTON-FAIRLEY, D., KOISTINEN, R. & 4 others (1990). Effect of insulin-like growth factor type I (IGF-I) and insulin on the secretion of sex hormone binding globulin and IGF-I binding protein (IBP-I) by human hepatoma cells. *J. Endocrinol.*, **124**, R1.

SYNDER, J.M. & D'ERCOLE, A.J. (1987). Somatomedin C insulin-like growth factor I production by human fetal lung tissue maintained *in vitro*. *Exp. Lung Res.*, **13**, 449.

SPORN, M.B. & TODARO, G.J. (1980). Autocrine secretion and malignant transformation of cells. *N. Engl. J. Med.*, **303**, 878.

STEGE, R., FROHLANDER, N., KJELL, C., POUSETTE, A. & VON SCHOULTZ, B. (1987). Steroid-sensitive proteins, proteins, growth hormone and somatomedin C in prostatic cancer: effects of parenteral and oral estrogen therapy. *Prostate*, **10**, 333.

STRACKE, M.L., ENGEL, J.D., WILSON, L.W., RECHLER, M.M., LIOTTA, L.A. & SCHIFFMANN, E. (1989). The type I insulin-like growth factor receptor is a motility receptor in human melanoma cells. *J. Biol. Chem.*, **264**, 21544.

SU, T.-S., LIU, W.-Y., HAN, S.-H. & 4 others (1989). Transcripts of the insulin-like growth factors I and II in human hepatoma. *Cancer Res.*, **49**, 1773.

SUZUKI, T., IWAFUCHI, M., YANAIHARA, C. & 5 others (1989). IGF-II like immunoreactivity in human tissues, neuroendocrine tumors and PC12 cells. *Diabetes Res. Clin. Prac.*, **7**, S21.

TALAVERA, F., REYNOLDS, R.K., ROBERTS, J.A. & MENON, K.M.J. (1990). Insulin-like growth factor I receptors in normal and neoplastic human endometrium. *Cancer Res.*, **50**, 3019.

THOMPSON, M.A., COX, A.J., WHITEHEAD, R.H. & JONAS, H.A. (1990). Autocrine regulation of human tumor cell proliferation by insulin-like growth factor II: an *in vitro* model. *Endocrinol.*, **126**, 3033.

TRICOLI, J.V., RALL, L.B., KARAKOUSIS, C.P. & 4 others (1986). Enhanced levels of insulin-like growth factor messenger RNA in human colon carcinomas and liposarcomas. *Cancer Res.*, **46**, 6169.

ULLRICH, A., GRAY, A., TAM, A.W. & 7 others (1986). Insulin-like growth factor I receptor primary structure: comparison with insulin receptor suggest structural determinants that define functional specificity. *EMBO J.*, **4**, 2503.

UNDERWOOD, L.E., D'ERCOLE, A.J., CLEMMONS, D.R. & VAN WYK, J.J. (1986). Paracrine functions of somatomedins. *Clin Endocrinol Metabol.*, **15**, 59.

VAN BUUL-OFFERS, S., VEDA, I. & VAN DEN BRANDE, J.L. (1986). Biosynthetic somatomedin-C increases the length and weight of Snell dwarf mice. *Pediatr. Res.*, **20**, 825.

VAN SCHRAVENDIJK, C.F.H., FORIERS, A., VAN DEN BRANDE, J.L. & PIPELEERS, D.G. (1987). Evidence of the presence of type I insulin-like growth factor receptors on rat pancreatic A and B cells. *Endocrinol.*, **121**, 1784.

VAN WYK, J.J. & UNDERWOOD, L.E. (1978). The somatomedins and their actions. In Litwack, G. (ed.), *Biochemical Actions of Hormones*. Academic Press Inc: New York, **5**, 101.

VAN WYK, J.J., GRAVES, D.C., CASELLA, S.J. & JACOBS, S. (1985). Evidence from monoclonal antibody studies that insulin stimulates dexoyribonucleic acid synthesis through the type I somatomedin receptor. *J. Clin. Endocrinol. Metabol.*, **61**, 639.

VERLAND, S. & GAMMELTOFT, S. (1989). Functional receptors for insulin-like growth factors I and II in rat thymocytes and mouse thymoma cells. *Mol. Cell Endocrinol.*, **67**, 207.

WEIMA, S.M., STET, L.H., VAN ROOIJEN, M.A. & 4 others (1989). Human teratocarcinoma cells express functional insulin-like growth factor I receptors. *Exp. Cell Res.*, **184**, 427.

WHITEHEAD, R.H., NOVAK, U., THOMAS, R.J., LUKEIS, R.E., WALKER, R.E. & JONES, J. (1989). A new gastric carcinoma cell line (LIM1839) derived from a young caucasian male. *Int. J. Cancer*, **44**, 1100.

WIDMER, U., ZAPF, J. & FROESCH, E.R. (1983). Is extrapancreatic tumour hypoglycaemia associated with elevated levels of IGFII? *J. Clin. Endocrinol. Metab.*, **55**, 833.

WIEDEMANN, H.R. (1964). Complexe malformatif familial avec hernie ombilicale et macroglossie. Un 'syndrome nouveau'. *J. Genet. Hum.*, **13**, 223.

WILLISON, K. (1991). Opposite imprinting of the mouse IGF2 and IGF2r genes. *Trends Genet.*, **7**, 107.

WYNNE WILLIAMS, D., DILLWYN, WILLIAMS, E. & WYNFORD-THOMAS, D. (1989). Evidence for autocrine production of IGF-I in human thyroid adenomas. *Mol. Cell. Endocrinol.*, **61**, 139.

YASHIRO, T., OHBA, Y., MURAKAMI, H. & 5 others (1989). Expression of insulin-like growth factor receptors in primary human thyroid neoplasms. *Acta Endocrinol.*, **121**, 112.

YEE, D., PAIK, S., LEBOVIC, G.S. & 5 others (1989*a*). Analysis of insulin-like growth factor I gene expression in malignancy: evidence for a paracrine role in human breast cancer. *Mol. Endocrinol.*, **3**, 509.

YEE, D., FAVONI, R.E., LUPU, R. & 8 others (1989*b*). The insulin-like growth factor binding protein BP25 is expressed by human breast cancer cells. *Biochem. Biophys. Res. Comm.*, **158**, 38.

YEE, D., FAVONI, R.E., LIPPMAN, M.E. & POWELL, D.R. (1991). Identification of insulin-like growth factor binding proteins in breast cancer cells. *Breast Cancer Res. Treat.*, **18**, 3.

ZAPF, J. & FROESCH, E.R. (1986). Insulin-like growth factors somatomedins: structure, secretion, biological actions and physiological role. *Hormone Res.*, **24**, 121.


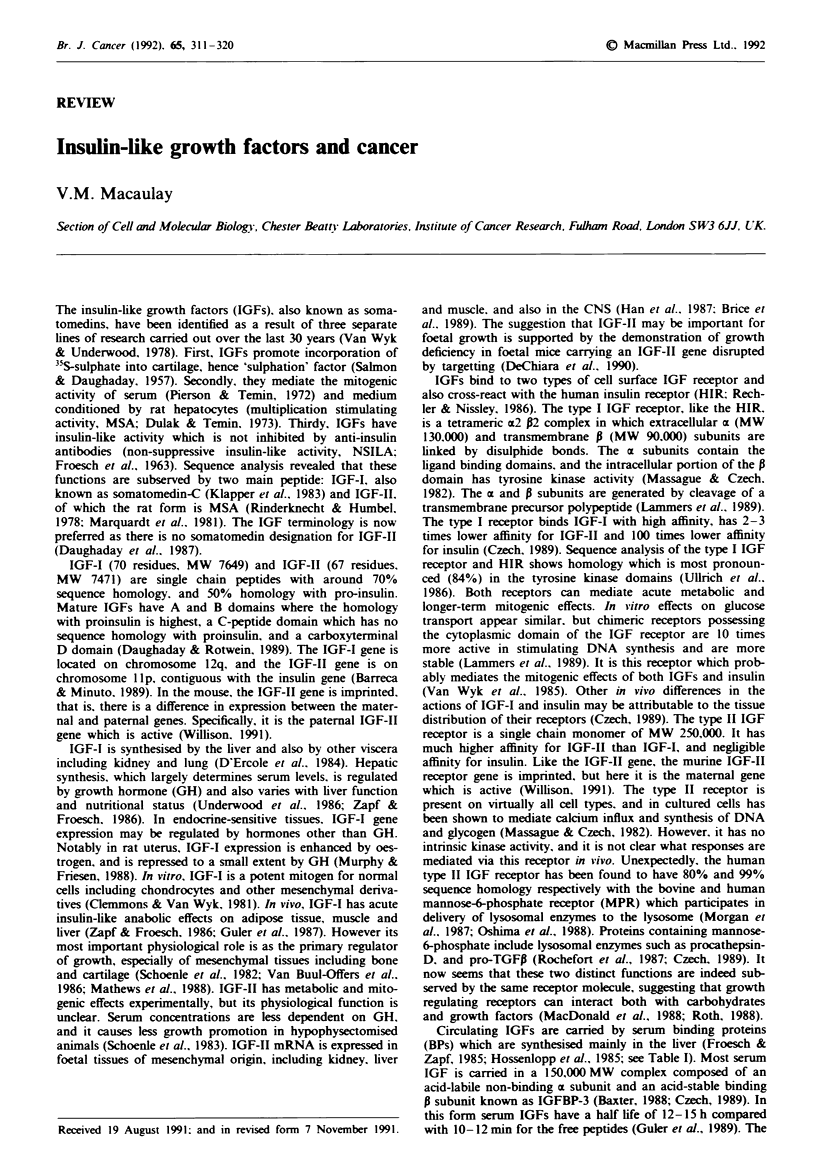

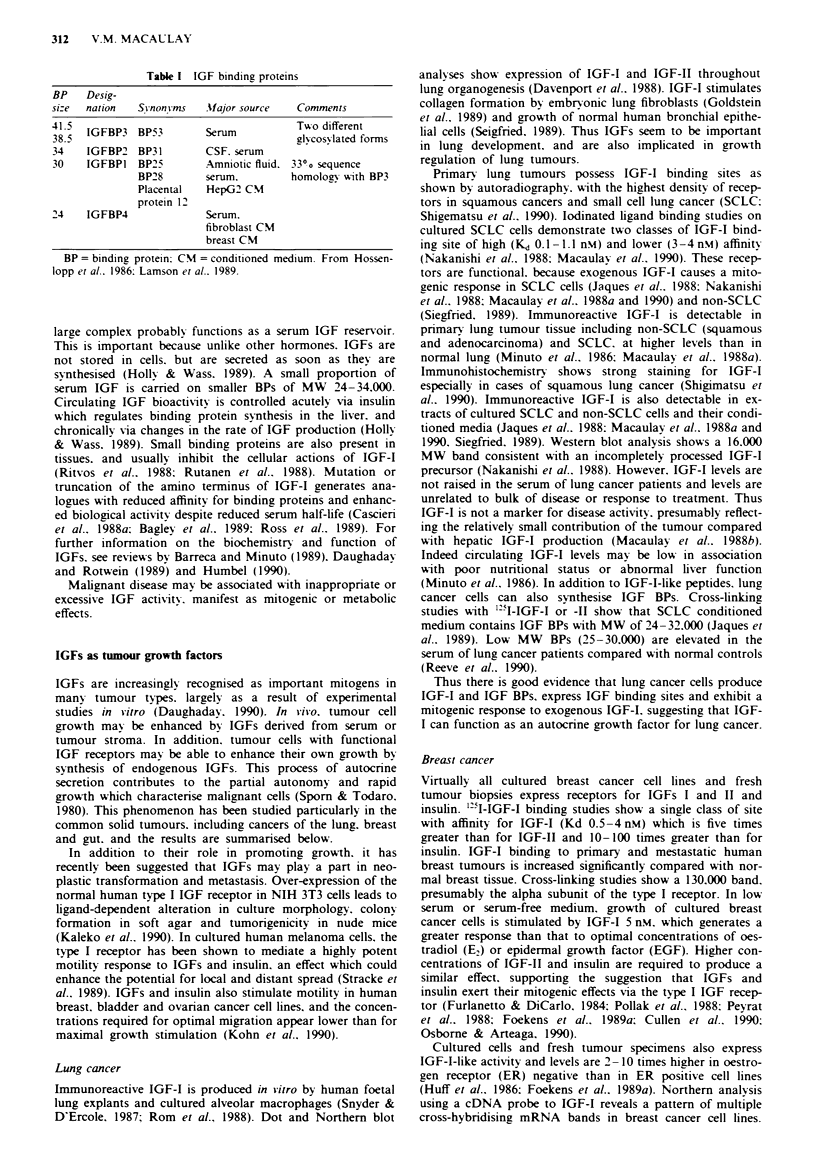

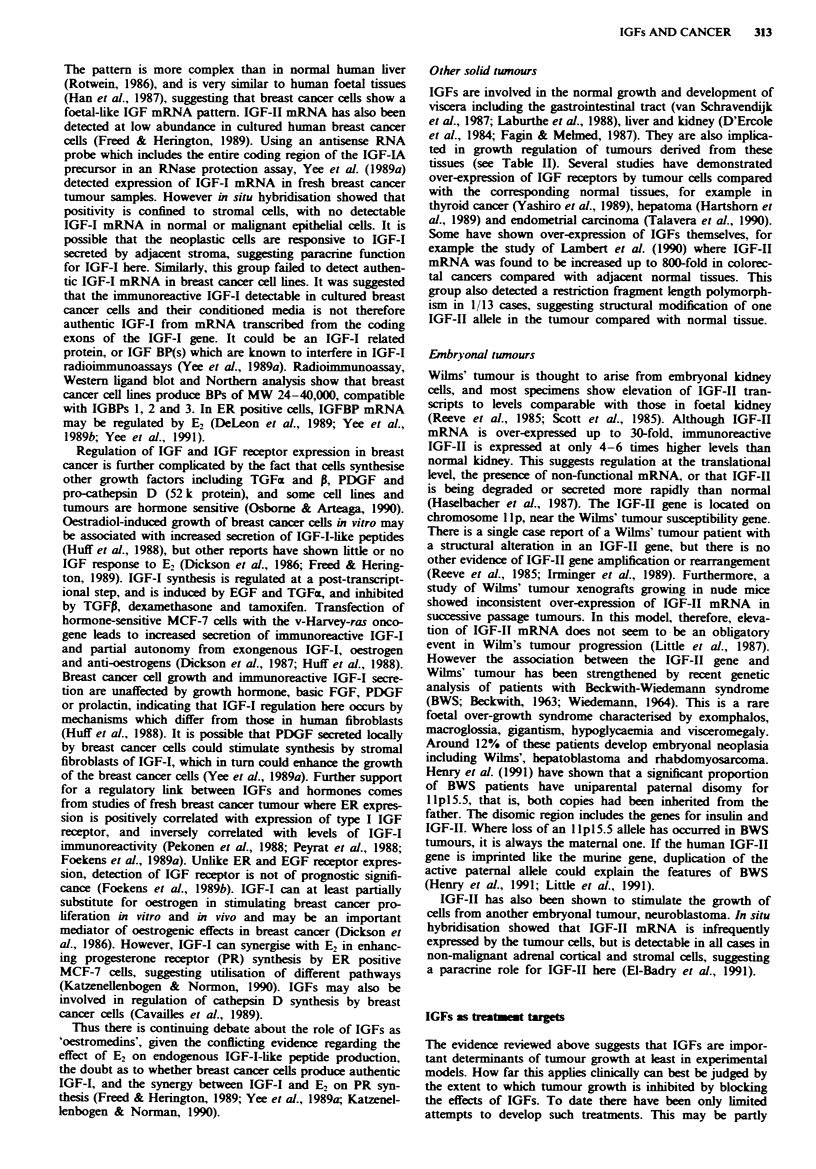

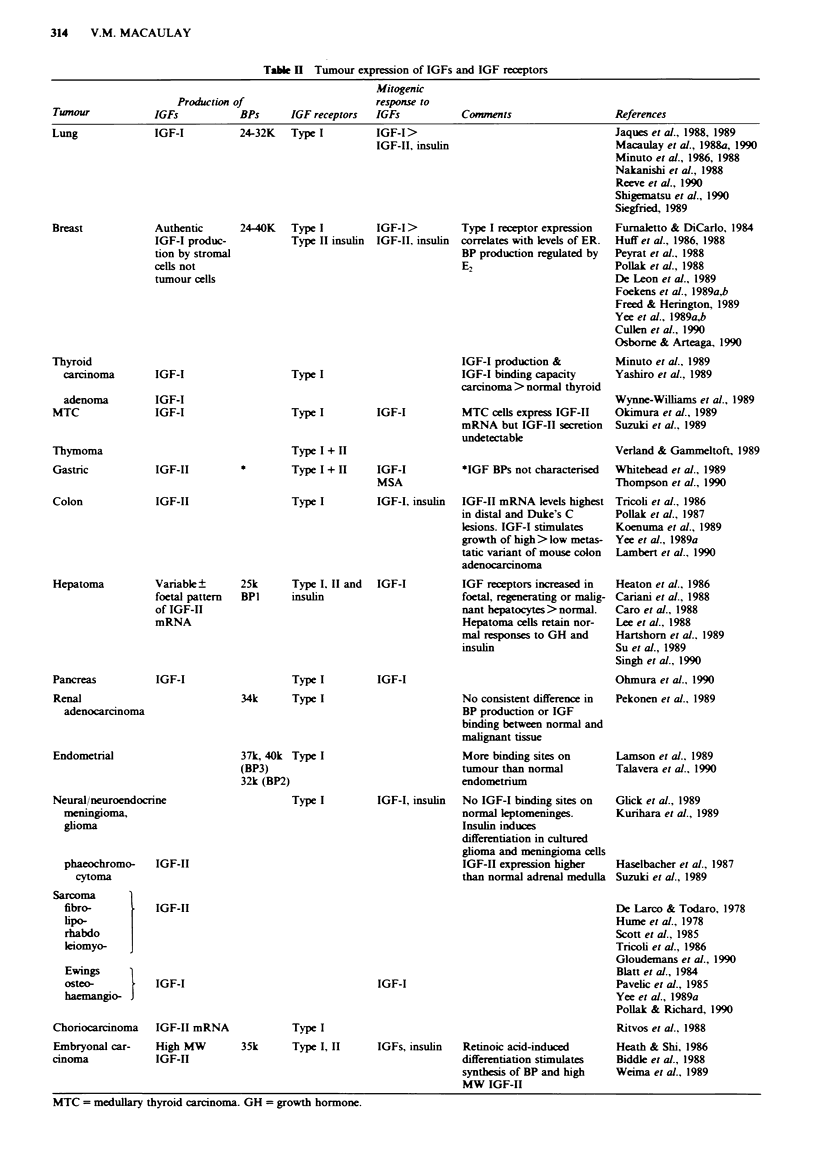

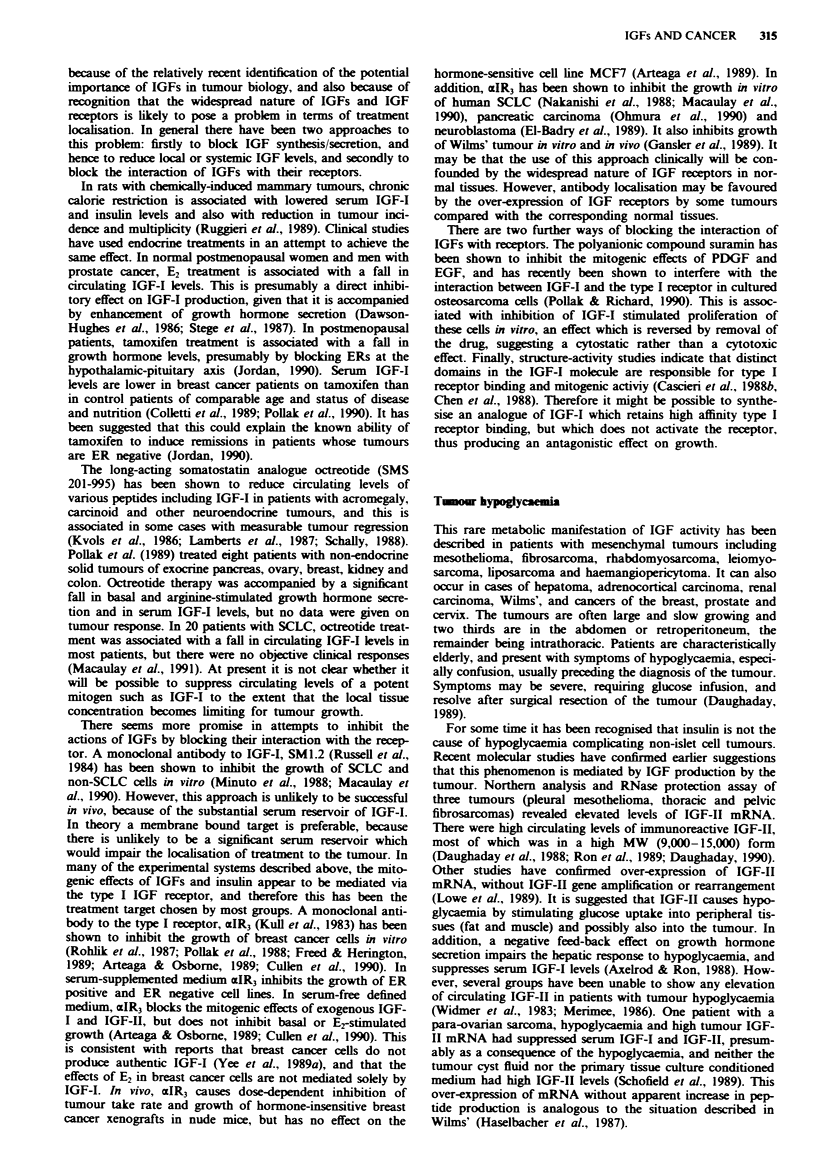

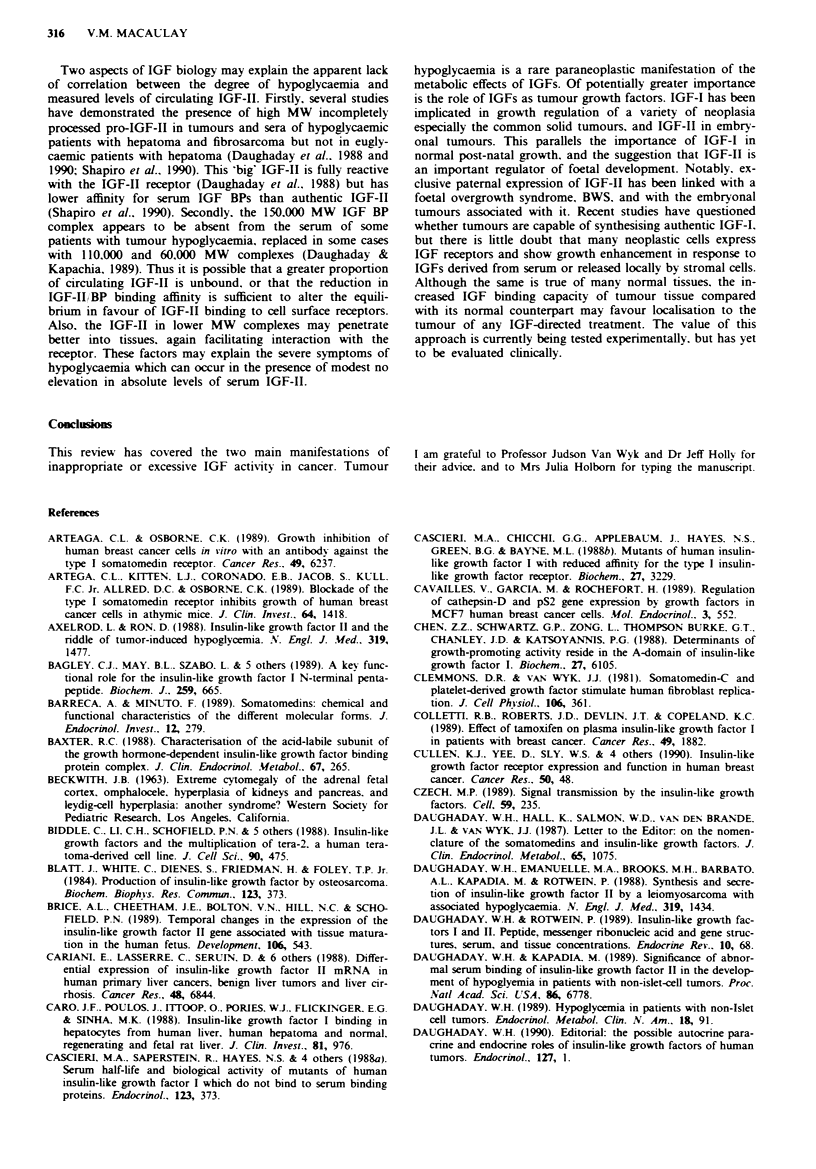

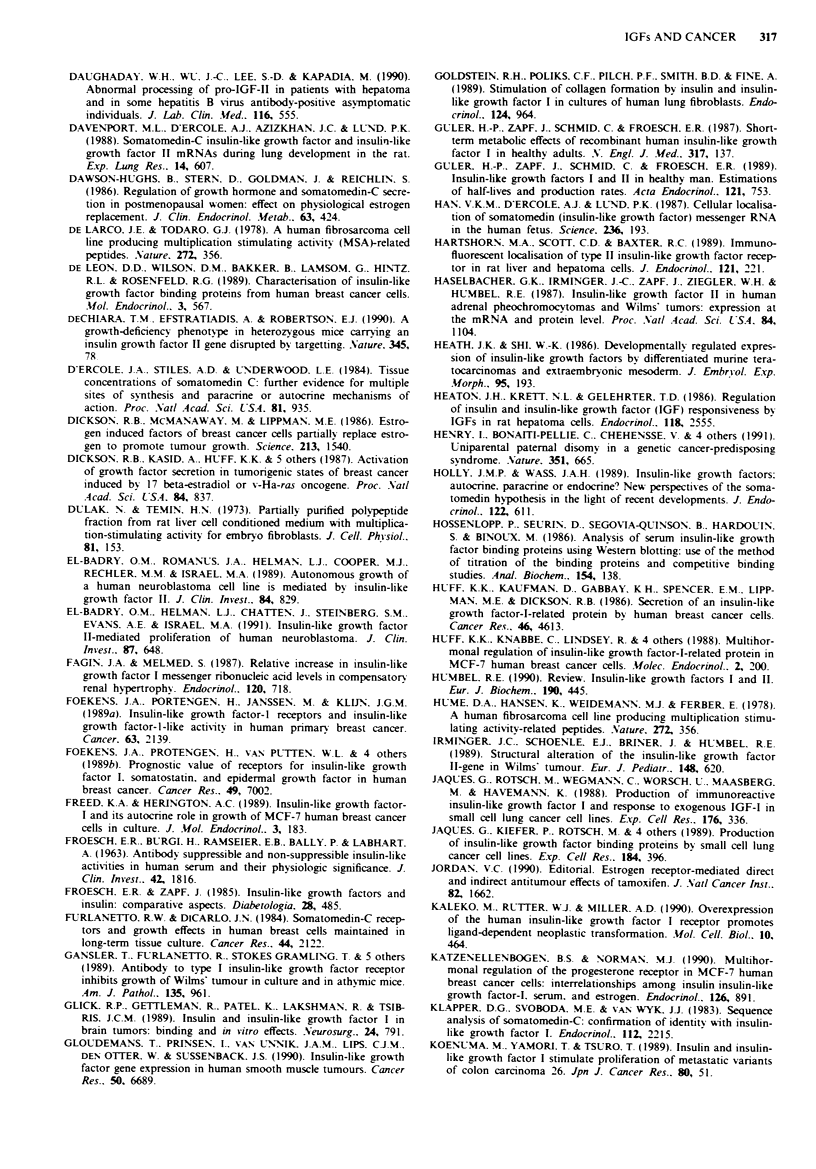

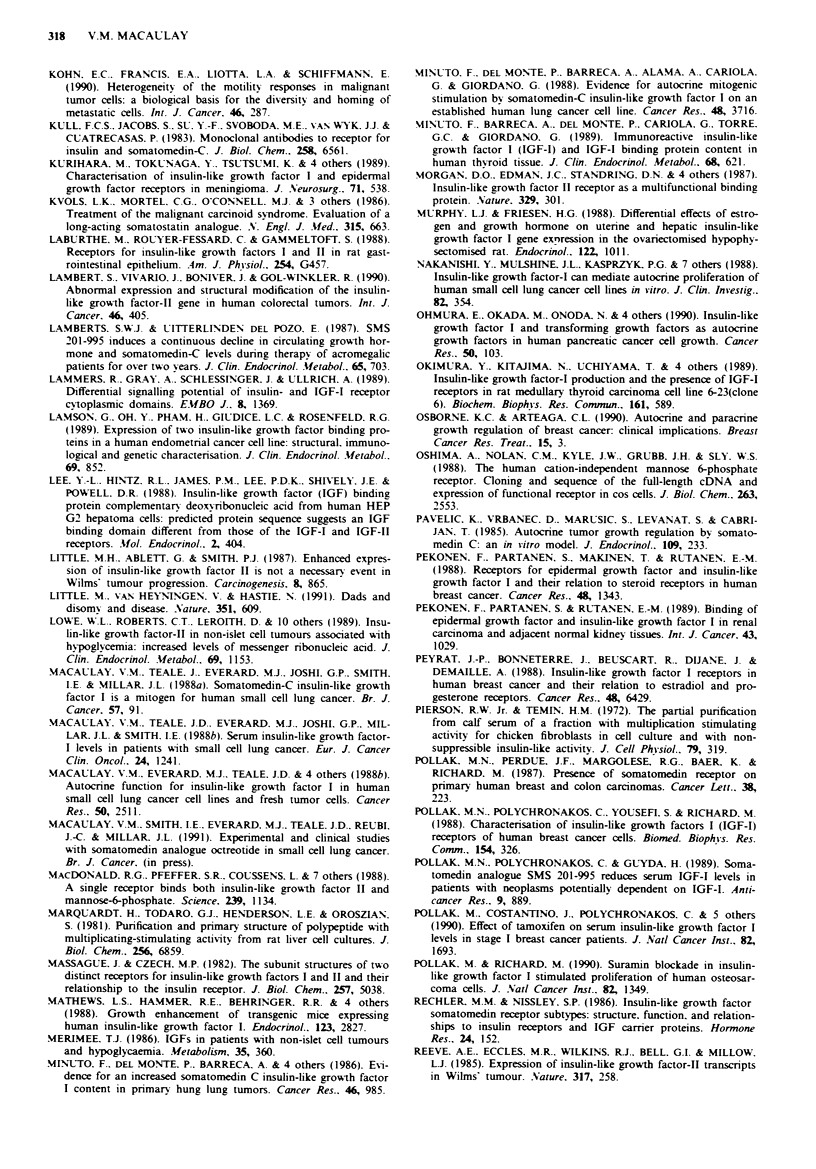

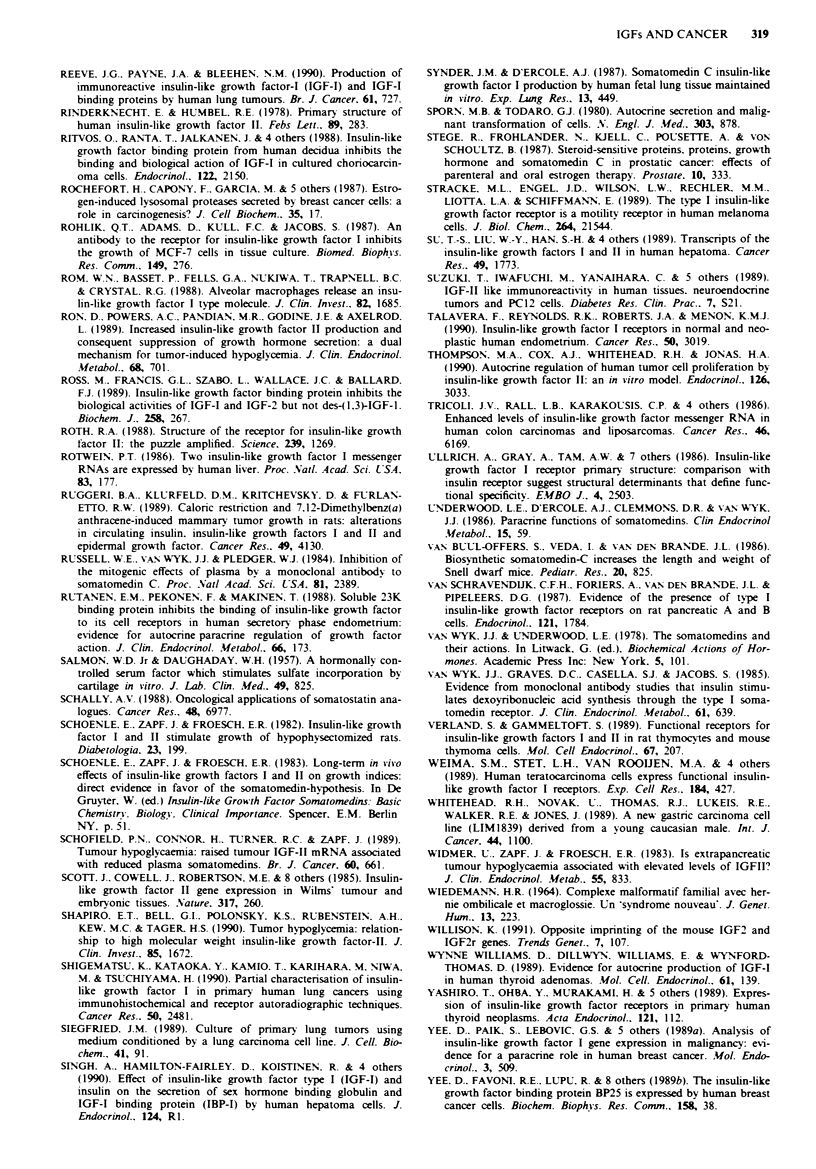

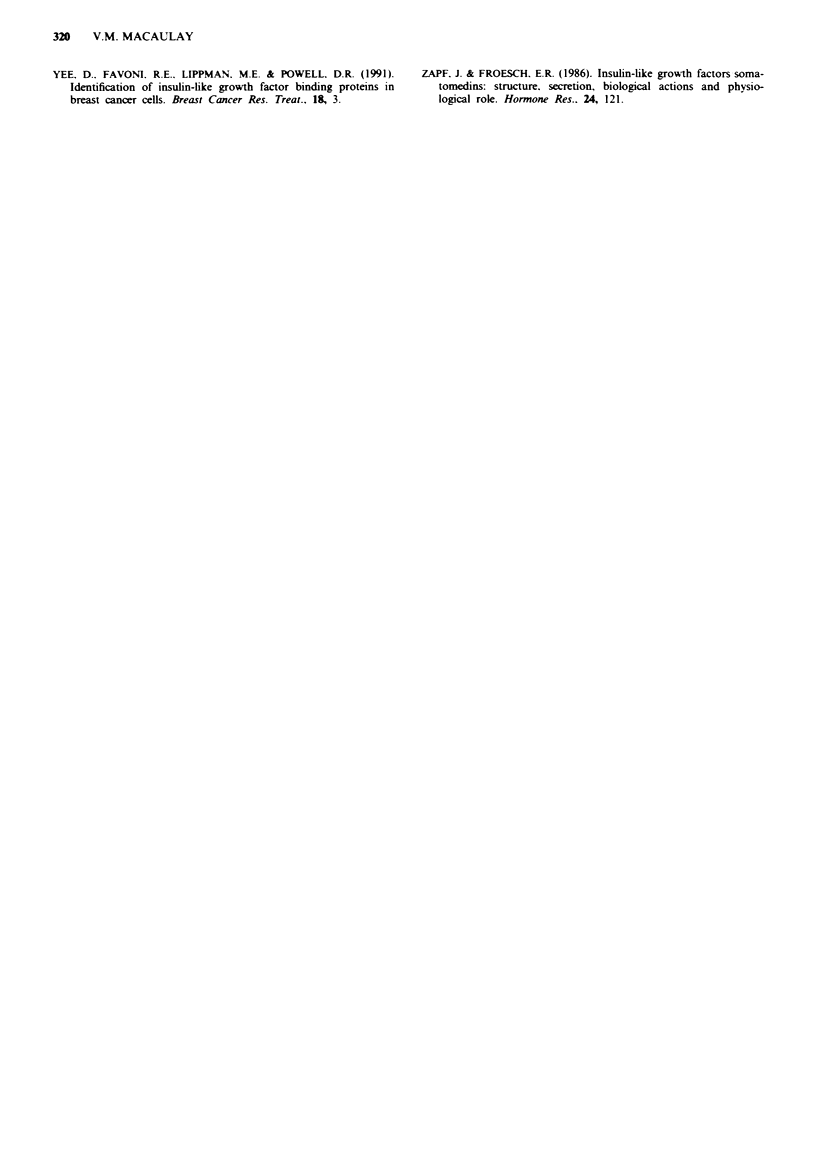


## References

[OCR_00974] Arteaga C. L., Kitten L. J., Coronado E. B., Jacobs S., Kull F. C., Allred D. C., Osborne C. K. (1989). Blockade of the type I somatomedin receptor inhibits growth of human breast cancer cells in athymic mice.. J Clin Invest.

[OCR_01114] Arteaga C. L., Kitten L. J., Coronado E. B., Jacobs S., Kull F. C., Allred D. C., Osborne C. K. (1989). Blockade of the type I somatomedin receptor inhibits growth of human breast cancer cells in athymic mice.. J Clin Invest.

[OCR_01109] Arteaga C. L., Osborne C. K. (1989). Growth inhibition of human breast cancer cells in vitro with an antibody against the type I somatomedin receptor.. Cancer Res.

[OCR_00969] Arteaga C. L., Osborne C. K. (1989). Growth inhibition of human breast cancer cells in vitro with an antibody against the type I somatomedin receptor.. Cancer Res.

[OCR_01122] Axelrod L., Ron D. (1988). Insulin-like growth factor II and the riddle of tumor-induced hypoglycemia.. N Engl J Med.

[OCR_00982] Axelrod L., Ron D. (1988). Insulin-like growth factor II and the riddle of tumor-induced hypoglycemia.. N Engl J Med.

[OCR_01127] Bagley C. J., May B. L., Szabo L., McNamara P. J., Ross M., Francis G. L., Ballard F. J., Wallace J. C. (1989). A key functional role for the insulin-like growth factor 1 N-terminal pentapeptide.. Biochem J.

[OCR_00987] Bagley C. J., May B. L., Szabo L., McNamara P. J., Ross M., Francis G. L., Ballard F. J., Wallace J. C. (1989). A key functional role for the insulin-like growth factor 1 N-terminal pentapeptide.. Biochem J.

[OCR_01130] Barreca A., Minuto F. (1989). Somatomedins: chemical and functional characteristics of the different molecular forms.. J Endocrinol Invest.

[OCR_00990] Barreca A., Minuto F. (1989). Somatomedins: chemical and functional characteristics of the different molecular forms.. J Endocrinol Invest.

[OCR_00995] Baxter R. C. (1988). Characterization of the acid-labile subunit of the growth hormone-dependent insulin-like growth factor binding protein complex.. J Clin Endocrinol Metab.

[OCR_01135] Baxter R. C. (1988). Characterization of the acid-labile subunit of the growth hormone-dependent insulin-like growth factor binding protein complex.. J Clin Endocrinol Metab.

[OCR_01146] Biddle C., Li C. H., Schofield P. N., Tate V. E., Hopkins B., Engstrom W., Huskisson N. S., Graham C. F. (1988). Insulin-like growth factors and the multiplication of Tera-2, a human teratoma-derived cell line.. J Cell Sci.

[OCR_01006] Biddle C., Li C. H., Schofield P. N., Tate V. E., Hopkins B., Engstrom W., Huskisson N. S., Graham C. F. (1988). Insulin-like growth factors and the multiplication of Tera-2, a human teratoma-derived cell line.. J Cell Sci.

[OCR_01151] Blatt J., White C., Dienes S., Friedman H., Foley T. P. (1984). Production of an insulin-like growth factor by osteosarcoma.. Biochem Biophys Res Commun.

[OCR_01011] Blatt J., White C., Dienes S., Friedman H., Foley T. P. (1984). Production of an insulin-like growth factor by osteosarcoma.. Biochem Biophys Res Commun.

[OCR_01018] Brice A. L., Cheetham J. E., Bolton V. N., Hill N. C., Schofield P. N. (1989). Temporal changes in the expression of the insulin-like growth factor II gene associated with tissue maturation in the human fetus.. Development.

[OCR_01158] Brice A. L., Cheetham J. E., Bolton V. N., Hill N. C., Schofield P. N. (1989). Temporal changes in the expression of the insulin-like growth factor II gene associated with tissue maturation in the human fetus.. Development.

[OCR_01164] Cariani E., Lasserre C., Seurin D., Hamelin B., Kemeny F., Franco D., Czech M. P., Ullrich A., Brechot C. (1988). Differential expression of insulin-like growth factor II mRNA in human primary liver cancers, benign liver tumors, and liver cirrhosis.. Cancer Res.

[OCR_01024] Cariani E., Lasserre C., Seurin D., Hamelin B., Kemeny F., Franco D., Czech M. P., Ullrich A., Brechot C. (1988). Differential expression of insulin-like growth factor II mRNA in human primary liver cancers, benign liver tumors, and liver cirrhosis.. Cancer Res.

[OCR_01170] Caro J. F., Poulos J., Ittoop O., Pories W. J., Flickinger E. G., Sinha M. K. (1988). Insulin-like growth factor I binding in hepatocytes from human liver, human hepatoma, and normal, regenerating, and fetal rat liver.. J Clin Invest.

[OCR_01030] Caro J. F., Poulos J., Ittoop O., Pories W. J., Flickinger E. G., Sinha M. K. (1988). Insulin-like growth factor I binding in hepatocytes from human liver, human hepatoma, and normal, regenerating, and fetal rat liver.. J Clin Invest.

[OCR_01043] Cascieri M. A., Chicchi G. G., Applebaum J., Hayes N. S., Green B. G., Bayne M. L. (1988). Mutants of human insulin-like growth factor I with reduced affinity for the type 1 insulin-like growth factor receptor.. Biochemistry.

[OCR_01183] Cascieri M. A., Chicchi G. G., Applebaum J., Hayes N. S., Green B. G., Bayne M. L. (1988). Mutants of human insulin-like growth factor I with reduced affinity for the type 1 insulin-like growth factor receptor.. Biochemistry.

[OCR_01036] Cascieri M. A., Saperstein R., Hayes N. S., Green B. G., Chicchi G. G., Applebaum J., Bayne M. L. (1988). Serum half-life and biological activity of mutants of human insulin-like growth factor I which do not bind to serum binding proteins.. Endocrinology.

[OCR_01176] Cascieri M. A., Saperstein R., Hayes N. S., Green B. G., Chicchi G. G., Applebaum J., Bayne M. L. (1988). Serum half-life and biological activity of mutants of human insulin-like growth factor I which do not bind to serum binding proteins.. Endocrinology.

[OCR_01046] Cavailles V., Garcia M., Rochefort H. (1989). Regulation of cathepsin-D and pS2 gene expression by growth factors in MCF7 human breast cancer cells.. Mol Endocrinol.

[OCR_01186] Cavailles V., Garcia M., Rochefort H. (1989). Regulation of cathepsin-D and pS2 gene expression by growth factors in MCF7 human breast cancer cells.. Mol Endocrinol.

[OCR_01191] Chen Z. Z., Schwartz G. P., Zong L., Burke G. T., Chanley J. D., Katsoyannis P. G. (1988). Determinants of growth-promoting activity reside in the A-domain of insulin-like growth factor I.. Biochemistry.

[OCR_01051] Chen Z. Z., Schwartz G. P., Zong L., Burke G. T., Chanley J. D., Katsoyannis P. G. (1988). Determinants of growth-promoting activity reside in the A-domain of insulin-like growth factor I.. Biochemistry.

[OCR_01057] Clemmons D. R., Van Wyk J. J. (1981). Somatomedin-C and platelet-derived growth factor stimulate human fibroblast replication.. J Cell Physiol.

[OCR_01197] Clemmons D. R., Van Wyk J. J. (1981). Somatomedin-C and platelet-derived growth factor stimulate human fibroblast replication.. J Cell Physiol.

[OCR_01062] Colletti R. B., Roberts J. D., Devlin J. T., Copeland K. C. (1989). Effect of tamoxifen on plasma insulin-like growth factor I in patients with breast cancer.. Cancer Res.

[OCR_01202] Colletti R. B., Roberts J. D., Devlin J. T., Copeland K. C. (1989). Effect of tamoxifen on plasma insulin-like growth factor I in patients with breast cancer.. Cancer Res.

[OCR_01212] Czech M. P. (1989). Signal transmission by the insulin-like growth factors.. Cell.

[OCR_01072] Czech M. P. (1989). Signal transmission by the insulin-like growth factors.. Cell.

[OCR_01284] D'Ercole A. J., Stiles A. D., Underwood L. E. (1984). Tissue concentrations of somatomedin C: further evidence for multiple sites of synthesis and paracrine or autocrine mechanisms of action.. Proc Natl Acad Sci U S A.

[OCR_01222] Daughaday W. H., Emanuele M. A., Brooks M. H., Barbato A. L., Kapadia M., Rotwein P. (1988). Synthesis and secretion of insulin-like growth factor II by a leiomyosarcoma with associated hypoglycemia.. N Engl J Med.

[OCR_01082] Daughaday W. H., Emanuele M. A., Brooks M. H., Barbato A. L., Kapadia M., Rotwein P. (1988). Synthesis and secretion of insulin-like growth factor II by a leiomyosarcoma with associated hypoglycemia.. N Engl J Med.

[OCR_01078] Daughaday W. H., Hall K., Salmon W. D., Van den Brande J. L., Van Wyk J. J. (1987). On the nomenclature of the somatomedins and insulin-like growth factors.. J Clin Endocrinol Metab.

[OCR_01218] Daughaday W. H., Hall K., Salmon W. D., Van den Brande J. L., Van Wyk J. J. (1987). On the nomenclature of the somatomedins and insulin-like growth factors.. J Clin Endocrinol Metab.

[OCR_01092] Daughaday W. H., Kapadia M. (1989). Significance of abnormal serum binding of insulin-like growth factor II in the development of hypoglycemia in patients with non-islet-cell tumors.. Proc Natl Acad Sci U S A.

[OCR_01232] Daughaday W. H., Kapadia M. (1989). Significance of abnormal serum binding of insulin-like growth factor II in the development of hypoglycemia in patients with non-islet-cell tumors.. Proc Natl Acad Sci U S A.

[OCR_01242] Daughaday W. H. (1990). The possible autocrine/paracrine and endocrine roles of insulin-like growth factors of human tumors.. Endocrinology.

[OCR_01251] Daughaday W. H., Wu J. C., Lee S. D., Kapadia M. (1990). Abnormal processing of pro-IGF-II in patients with hepatoma and in some hepatitis B virus antibody-positive asymptomatic individuals.. J Lab Clin Med.

[OCR_01257] Davenport M. L., D'Ercole A. J., Azizkhan J. C., Lund P. K. (1988). Somatomedin-C/insulinlike growth factor I (Sm-C/IGF-I) and insulinlike growth factor II (IGF-II) mRNAs during lung development in the rat.. Exp Lung Res.

[OCR_01263] Dawson-Hughes B., Stern D., Goldman J., Reichlin S. (1986). Regulation of growth hormone and somatomedin-C secretion in postmenopausal women: effect of physiological estrogen replacement.. J Clin Endocrinol Metab.

[OCR_01269] De Larco J. E., Tadaro G. J. (1978). A human fibrosarcoma cell line producing multiplication stimulating activity (MSA)-related peptides.. Nature.

[OCR_01274] De Leon D. D., Wilson D. M., Bakker B., Lamsom G., Hintz R. L., Rosenfeld R. G. (1989). Characterization of insulin-like growth factor binding proteins from human breast cancer cells.. Mol Endocrinol.

[OCR_01297] Dickson R. B., Kasid A., Huff K. K., Bates S. E., Knabbe C., Bronzert D., Gelmann E. P., Lippman M. E. (1987). Activation of growth factor secretion in tumorigenic states of breast cancer induced by 17 beta-estradiol or v-Ha-ras oncogene.. Proc Natl Acad Sci U S A.

[OCR_01290] Dickson R. B., McManaway M. E., Lippman M. E. (1986). Estrogen-induced factors of breast cancer cells partially replace estrogen to promote tumor growth.. Science.

[OCR_01303] Dulak N. C., Temin H. M. (1973). A partially purified polypeptide fraction from rat liver cell conditioned medium with multiplication-stimulating activity for embryo fibroblasts.. J Cell Physiol.

[OCR_01316] El-Badry O. M., Helman L. J., Chatten J., Steinberg S. M., Evans A. E., Israel M. A. (1991). Insulin-like growth factor II-mediated proliferation of human neuroblastoma.. J Clin Invest.

[OCR_01310] El-Badry O. M., Romanus J. A., Helman L. J., Cooper M. J., Rechler M. M., Israel M. A. (1989). Autonomous growth of a human neuroblastoma cell line is mediated by insulin-like growth factor II.. J Clin Invest.

[OCR_01343] FROESCH E. R., BUERGI H., RAMSEIER E. B., BALLY P., LABHART A. (1963). ANTIBODY-SUPPRESSIBLE AND NONSUPPRESSIBLE INSULIN-LIKE ACTIVITIES IN HUMAN SERUM AND THEIR PHYSIOLOGIC SIGNIFICANCE. AN INSULIN ASSAY WITH ADIPOSE TISSUE OF INCREASED PRECISION AND SPECIFICITY.. J Clin Invest.

[OCR_01321] Fagin J. A., Melmed S. (1987). Relative increase in insulin-like growth factor I messenger ribonucleic acid levels in compensatory renal hypertrophy.. Endocrinology.

[OCR_01326] Foekens J. A., Portengen H., Janssen M., Klijn J. G. (1989). Insulin-like growth factor-1 receptors and insulin-like growth factor-1-like activity in human primary breast cancer.. Cancer.

[OCR_01338] Freed K. A., Herington A. C. (1989). Insulin-like growth factor-I and its autocrine role in growth of MCF-7 human breast cancer cells in culture.. J Mol Endocrinol.

[OCR_01349] Froesch E. R., Zapf J. (1985). Insulin-like growth factors and insulin: comparative aspects.. Diabetologia.

[OCR_01353] Furlanetto R. W., DiCarlo J. N. (1984). Somatomedin-C receptors and growth effects in human breast cells maintained in long-term tissue culture.. Cancer Res.

[OCR_01356] Gansler T., Furlanetto R., Gramling T. S., Robinson K. A., Blocker N., Buse M. G., Sens D. A., Garvin A. J. (1989). Antibody to type I insulinlike growth factor receptor inhibits growth of Wilms' tumor in culture and in athymic mice.. Am J Pathol.

[OCR_01362] Glick R. P., Gettleman R., Patel K., Lakshman R., Tsibris J. C. (1989). Insulin and insulin-like growth factor I in brain tumors: binding and in vitro effects.. Neurosurgery.

[OCR_01369] Gloudemans T., Prinsen I., Van Unnik J. A., Lips C. J., Den Otter W., Sussenbach J. S. (1990). Insulin-like growth factor gene expression in human smooth muscle tumors.. Cancer Res.

[OCR_01372] Goldstein R. H., Poliks C. F., Pilch P. F., Smith B. D., Fine A. (1989). Stimulation of collagen formation by insulin and insulin-like growth factor I in cultures of human lung fibroblasts.. Endocrinology.

[OCR_01380] Guler H. P., Zapf J., Froesch E. R. (1987). Short-term metabolic effects of recombinant human insulin-like growth factor I in healthy adults.. N Engl J Med.

[OCR_01385] Guler H. P., Zapf J., Schmid C., Froesch E. R. (1989). Insulin-like growth factors I and II in healthy man. Estimations of half-lives and production rates.. Acta Endocrinol (Copenh).

[OCR_01388] Han V. K., D'Ercole A. J., Lund P. K. (1987). Cellular localization of somatomedin (insulin-like growth factor) messenger RNA in the human fetus.. Science.

[OCR_01393] Hartshorn M. A., Scott C. D., Baxter R. C. (1989). Immunofluorescent localization of type II insulin-like growth factor receptor in rat liver and hepatoma cells.. J Endocrinol.

[OCR_01400] Haselbacher G. K., Irminger J. C., Zapf J., Ziegler W. H., Humbel R. E. (1987). Insulin-like growth factor II in human adrenal pheochromocytomas and Wilms tumors: expression at the mRNA and protein level.. Proc Natl Acad Sci U S A.

[OCR_01405] Heath J. K., Shi W. K. (1986). Developmentally regulated expression of insulin-like growth factors by differentiated murine teratocarcinomas and extraembryonic mesoderm.. J Embryol Exp Morphol.

[OCR_01411] Heaton J. H., Krett N. L., Gelehrter T. D. (1986). Regulation of insulin and insulin-like growth factor (IGF) responsiveness by IGFs in rat hepatoma cells.. Endocrinology.

[OCR_01418] Henry I., Bonaiti-Pellié C., Chehensse V., Beldjord C., Schwartz C., Utermann G., Junien C. (1991). Uniparental paternal disomy in a genetic cancer-predisposing syndrome.. Nature.

[OCR_01423] Holly J. M., Wass J. A. (1989). Insulin-like growth factors; autocrine, paracrine or endocrine? New perspectives of the somatomedin hypothesis in the light of recent developments.. J Endocrinol.

[OCR_01429] Hossenlopp P., Seurin D., Segovia-Quinson B., Hardouin S., Binoux M. (1986). Analysis of serum insulin-like growth factor binding proteins using western blotting: use of the method for titration of the binding proteins and competitive binding studies.. Anal Biochem.

[OCR_01437] Huff K. K., Kaufman D., Gabbay K. H., Spencer E. M., Lippman M. E., Dickson R. B. (1986). Secretion of an insulin-like growth factor-I-related protein by human breast cancer cells.. Cancer Res.

[OCR_01440] Huff K. K., Knabbe C., Lindsey R., Kaufman D., Bronzert D., Lippman M. E., Dickson R. B. (1988). Multihormonal regulation of insulin-like growth factor-I-related protein in MCF-7 human breast cancer cells.. Mol Endocrinol.

[OCR_01445] Humbel R. E. (1990). Insulin-like growth factors I and II.. Eur J Biochem.

[OCR_01454] Irminger J. C., Schoenle E. J., Briner J., Humbel R. E. (1989). Structural alteration of the insulin-like growth factor II-gene in Wilms tumour.. Eur J Pediatr.

[OCR_01465] Jaques G., Kiefer P., Rotsch M., Hennig C., Göke R., Richter G., Havemann K. (1989). Production of insulin-like growth factor binding proteins by small-cell lung cancer cell lines.. Exp Cell Res.

[OCR_01459] Jaques G., Rotsch M., Wegmann C., Worsch U., Maasberg M., Havemann K. (1988). Production of immunoreactive insulin-like growth factor I and response to exogenous IGF-I in small cell lung cancer cell lines.. Exp Cell Res.

[OCR_01472] Jordan V. C. (1990). Estrogen receptor-mediated direct and indirect antitumor effects of tamoxifen.. J Natl Cancer Inst.

[OCR_01477] Kaleko M., Rutter W. J., Miller A. D. (1990). Overexpression of the human insulinlike growth factor I receptor promotes ligand-dependent neoplastic transformation.. Mol Cell Biol.

[OCR_01481] Katzenellenbogen B. S., Norman M. J. (1990). Multihormonal regulation of the progesterone receptor in MCF-7 human breast cancer cells: interrelationships among insulin/insulin-like growth factor-I, serum, and estrogen.. Endocrinology.

[OCR_01489] Klapper D. G., Svoboda M. E., Van Wyk J. J. (1983). Sequence analysis of somatomedin-C: confirmation of identity with insulin-like growth factor I.. Endocrinology.

[OCR_01501] Kohn E. C., Francis E. A., Liotta L. A., Schiffmann E. (1990). Heterogeneity of the motility responses in malignant tumor cells: a biological basis for the diversity and homing of metastatic cells.. Int J Cancer.

[OCR_01505] Kull F. C., Jacobs S., Su Y. F., Svoboda M. E., Van Wyk J. J., Cuatrecasas P. (1983). Monoclonal antibodies to receptors for insulin and somatomedin-C.. J Biol Chem.

[OCR_01510] Kurihara M., Tokunaga Y., Tsutsumi K., Kawaguchi T., Shigematsu K., Niwa M., Mori K. (1989). Characterization of insulin-like growth factor I and epidermal growth factor receptors in meningioma.. J Neurosurg.

[OCR_01514] Kvols L. K., Moertel C. G., O'Connell M. J., Schutt A. J., Rubin J., Hahn R. G. (1986). Treatment of the malignant carcinoid syndrome. Evaluation of a long-acting somatostatin analogue.. N Engl J Med.

[OCR_01531] Lamberts S. W., Uitterlinden P., del Pozo E. (1987). SMS 201-995 induces a continuous decline in circulating growth hormone and somatomedin-C levels during therapy of acromegalic patients for over two years.. J Clin Endocrinol Metab.

[OCR_01536] Lammers R., Gray A., Schlessinger J., Ullrich A. (1989). Differential signalling potential of insulin- and IGF-1-receptor cytoplasmic domains.. EMBO J.

[OCR_01541] Lamson G., Oh Y., Pham H., Giudice L. C., Rosenfeld R. G. (1989). Expression of two insulin-like growth factor-binding proteins in a human endometrial cancer cell line: structural, immunological, and genetic characterization.. J Clin Endocrinol Metab.

[OCR_01549] Lee Y. L., Hintz R. L., James P. M., Lee P. D., Shively J. E., Powell D. R. (1988). Insulin-like growth factor (IGF) binding protein complementary deoxyribonucleic acid from human HEP G2 hepatoma cells: predicted protein sequence suggests an IGF binding domain different from those of the IGF-I and IGF-II receptors.. Mol Endocrinol.

[OCR_01556] Little M. H., Ablett G., Smith P. J. (1987). Enhanced expression of insulin-like growth factor II is not a necessary event in Wilms' tumour progression.. Carcinogenesis.

[OCR_01559] Little M., Van Heyningen V., Hastie N. (1991). Dads and disomy and disease.. Nature.

[OCR_01593] MacDonald R. G., Pfeffer S. R., Coussens L., Tepper M. A., Brocklebank C. M., Mole J. E., Anderson J. K., Chen E., Czech M. P., Ullrich A. (1988). A single receptor binds both insulin-like growth factor II and mannose-6-phosphate.. Science.

[OCR_01583] Macaulay V. M., Everard M. J., Teale J. D., Trott P. A., Van Wyk J. J., Smith I. E., Millar J. L. (1990). Autocrine function for insulin-like growth factor I in human small cell lung cancer cell lines and fresh tumor cells.. Cancer Res.

[OCR_01577] Macaulay V. M., Teale J. D., Everard M. J., Joshi G. P., Millar J. L., Smith I. E. (1988). Serum insulin-like growth factor-I levels in patients with small cell lung cancer.. Eur J Cancer Clin Oncol.

[OCR_01598] Marquardt H., Todaro G. J., Henderson L. E., Oroszlan S. (1981). Purification and primary structure of a polypeptide with multiplication-stimulating activity from rat liver cell cultures. Homology with human insulin-like growth factor II.. J Biol Chem.

[OCR_01604] Massagué J., Czech M. P. (1982). The subunit structures of two distinct receptors for insulin-like growth factors I and II and their relationship to the insulin receptor.. J Biol Chem.

[OCR_01631] Minuto F., Barreca A., Del Monte P., Cariola G., Torre G. C., Giordano G. (1989). Immunoreactive insulin-like growth factor I (IGF-I) and IGF-I-binding protein content in human thyroid tissue.. J Clin Endocrinol Metab.

[OCR_01623] Minuto F., Del Monte P., Barreca A., Alama A., Cariola G., Giordano G. (1988). Evidence for autocrine mitogenic stimulation by somatomedin-C/insulin-like growth factor I on an established human lung cancer cell line.. Cancer Res.

[OCR_01618] Minuto F., Del Monte P., Barreca A., Fortini P., Cariola G., Catrambone G., Giordano G. (1986). Evidence for an increased somatomedin-C/insulin-like growth factor I content in primary human lung tumors.. Cancer Res.

[OCR_01636] Morgan D. O., Edman J. C., Standring D. N., Fried V. A., Smith M. C., Roth R. A., Rutter W. J. (1987). Insulin-like growth factor II receptor as a multifunctional binding protein.. Nature.

[OCR_01647] Nakanishi Y., Mulshine J. L., Kasprzyk P. G., Natale R. B., Maneckjee R., Avis I., Treston A. M., Gazdar A. F., Minna J. D., Cuttitta F. (1988). Insulin-like growth factor-I can mediate autocrine proliferation of human small cell lung cancer cell lines in vitro.. J Clin Invest.

[OCR_01653] Ohmura E., Okada M., Onoda N., Kamiya Y., Murakami H., Tsushima T., Shizume K. (1990). Insulin-like growth factor I and transforming growth factor alpha as autocrine growth factors in human pancreatic cancer cell growth.. Cancer Res.

[OCR_01659] Okimura Y., Kitajima N., Uchiyama T., Yagi H., Abe H., Shakutsui S., Chihara K. (1989). Insulin-like growth factor I (IGF-I) production and the presence of IGF-I receptors in rat medullary thyroid carcinoma cell line 6-23 (clone 6).. Biochem Biophys Res Commun.

[OCR_01670] Oshima A., Nolan C. M., Kyle J. W., Grubb J. H., Sly W. S. (1988). The human cation-independent mannose 6-phosphate receptor. Cloning and sequence of the full-length cDNA and expression of functional receptor in COS cells.. J Biol Chem.

[OCR_01675] Pavelić K., Vrbanec D., Marusić S., Levanat S., Cabrijan T. (1986). Autocrine tumour growth regulation by somatomedin C: an in-vitro model.. J Endocrinol.

[OCR_01680] Pekonen F., Partanen S., Mäkinen T., Rutanen E. M. (1988). Receptors for epidermal growth factor and insulin-like growth factor I and their relation to steroid receptors in human breast cancer.. Cancer Res.

[OCR_01686] Pekonen F., Partanen S., Rutanen E. M. (1989). Binding of epidermal growth factor and insulin-like growth-factor I in renal carcinoma and adjacent normal kidney tissue.. Int J Cancer.

[OCR_01694] Peyrat J. P., Bonneterre J., Beuscart R., Djiane J., Demaille A. (1988). Insulin-like growth factor 1 receptors in human breast cancer and their relation to estradiol and progesterone receptors.. Cancer Res.

[OCR_01700] Pierson R. W., Temin H. M. (1972). The partial purification from calf serum of a fraction with multiplication-stimulating activity for chicken fibroblasts in cell culture and with non-suppressible insulin-like activity.. J Cell Physiol.

[OCR_01706] Pollak M. N., Perdue J. F., Margolese R. G., Baer K., Richard M. (1987). Presence of somatomedin receptors on primary human breast and colon carcinomas.. Cancer Lett.

[OCR_01718] Pollak M. N., Polychronakos C., Guyda H. (1989). Somatostatin analogue SMS 201-995 reduces serum IGF-I levels in patients with neoplasms potentially dependent on IGF-I.. Anticancer Res.

[OCR_01712] Pollak M. N., Polychronakos C., Yousefi S., Richard M. (1988). Characterization of insulin-like growth factor I (IGF-I) receptors of human breast cancer cells.. Biochem Biophys Res Commun.

[OCR_01724] Pollak M., Costantino J., Polychronakos C., Blauer S. A., Guyda H., Redmond C., Fisher B., Margolese R. (1990). Effect of tamoxifen on serum insulinlike growth factor I levels in stage I breast cancer patients.. J Natl Cancer Inst.

[OCR_01728] Pollak M., Richard M. (1990). Suramin blockade of insulinlike growth factor I-stimulated proliferation of human osteosarcoma cells.. J Natl Cancer Inst.

[OCR_01735] Rechler M. M., Nissley S. P. (1986). Insulin-like growth factor (IGF)/somatomedin receptor subtypes: structure, function, and relationships to insulin receptors and IGF carrier proteins.. Horm Res.

[OCR_01741] Reeve A. E., Eccles M. R., Wilkins R. J., Bell G. I., Millow L. J. (1985). Expression of insulin-like growth factor-II transcripts in Wilms' tumour.. Nature.

[OCR_01746] Reeve J. G., Payne J. A., Bleehen N. M. (1990). Production of immunoreactive insulin-like growth factor-I (IGF-I) and IGF-I binding proteins by human lung tumours.. Br J Cancer.

[OCR_01752] Rinderknecht E., Humbel R. E. (1978). Primary structure of human insulin-like growth factor II.. FEBS Lett.

[OCR_01756] Ritvos O., Ranta T., Jalkanen J., Suikkari A. M., Voutilainen R., Bohn H., Rutanen E. M. (1988). Insulin-like growth factor (IGF) binding protein from human decidua inhibits the binding and biological action of IGF-I in cultured choriocarcinoma cells.. Endocrinology.

[OCR_01767] Rohlik Q. T., Adams D., Kull F. C., Jacobs S. (1987). An antibody to the receptor for insulin-like growth factor I inhibits the growth of MCF-7 cells in tissue culture.. Biochem Biophys Res Commun.

[OCR_01773] Rom W. N., Basset P., Fells G. A., Nukiwa T., Trapnell B. C., Crysal R. G. (1988). Alveolar macrophages release an insulin-like growth factor I-type molecule.. J Clin Invest.

[OCR_01775] Ron D., Powers A. C., Pandian M. R., Godine J. E., Axelrod L. (1989). Increased insulin-like growth factor II production and consequent suppression of growth hormone secretion: a dual mechanism for tumor-induced hypoglycemia.. J Clin Endocrinol Metab.

[OCR_01782] Ross M., Francis G. L., Szabo L., Wallace J. C., Ballard F. J. (1989). Insulin-like growth factor (IGF)-binding proteins inhibit the biological activities of IGF-1 and IGF-2 but not des-(1-3)-IGF-1.. Biochem J.

[OCR_01788] Roth R. A. (1988). Structure of the receptor for insulin-like growth factor II: the puzzle amplified.. Science.

[OCR_01799] Ruggeri B. A., Klurfeld D. M., Kritchevsky D., Furlanetto R. W. (1989). Caloric restriction and 7,12-dimethylbenz(a)anthracene-induced mammary tumor growth in rats: alterations in circulating insulin, insulin-like growth factors I and II, and epidermal growth factor.. Cancer Res.

[OCR_01806] Russell W. E., Van Wyk J. J., Pledger W. J. (1984). Inhibition of the mitogenic effects of plasma by a monoclonal antibody to somatomedin C.. Proc Natl Acad Sci U S A.

[OCR_01809] Rutanen E. M., Pekonen F., Mäkinen T. (1988). Soluble 34K binding protein inhibits the binding of insulin-like growth factor I to its cell receptors in human secretory phase endometrium: evidence for autocrine/paracrine regulation of growth factor action.. J Clin Endocrinol Metab.

[OCR_01816] SALMON W. D., DAUGHADAY W. H. (1957). A hormonally controlled serum factor which stimulates sulfate incorporation by cartilage in vitro.. J Lab Clin Med.

[OCR_01823] Schally A. V. (1988). Oncological applications of somatostatin analogues.. Cancer Res.

[OCR_01838] Schofield P. N., Connor H., Turner R. C., Zapf J. (1989). Tumour hypoglycaemia: raised tumour IGFII mRNA associated with reduced plasma somatomedins.. Br J Cancer.

[OCR_01845] Scott J., Cowell J., Robertson M. E., Priestley L. M., Wadey R., Hopkins B., Pritchard J., Bell G. I., Rall L. B., Graham C. F. (1985). Insulin-like growth factor-II gene expression in Wilms' tumour and embryonic tissues.. Nature.

[OCR_01850] Shapiro E. T., Bell G. I., Polonsky K. S., Rubenstein A. H., Kew M. C., Tager H. S. (1990). Tumor hypoglycemia: relationship to high molecular weight insulin-like growth factor-II.. J Clin Invest.

[OCR_01857] Shigematsu K., Kataoka Y., Kamio T., Kurihara M., Niwa M., Tsuchiyama H. (1990). Partial characterization of insulin-like growth factor I in primary human lung cancers using immunohistochemical and receptor autoradiographic techniques.. Cancer Res.

[OCR_01873] Snyder J. M., D'Ercole A. J. (1987). Somatomedin C/insulin-like growth factor I production by human fetal lung tissue maintained in vitro.. Exp Lung Res.

[OCR_01878] Sporn M. B., Todaro G. J. (1980). Autocrine secretion and malignant transformation of cells.. N Engl J Med.

[OCR_01882] Stege R., Fröhlander N., Carlström K., Pousette A., von Schoultz B. (1987). Steroid-sensitive proteins, growth hormone and somatomedin C in prostatic cancer: effects of parenteral and oral estrogen therapy.. Prostate.

[OCR_01888] Stracke M. L., Engel J. D., Wilson L. W., Rechler M. M., Liotta L. A., Schiffmann E. (1989). The type I insulin-like growth factor receptor is a motility receptor in human melanoma cells.. J Biol Chem.

[OCR_01896] Su T. S., Liu W. Y., Han S. H., Jansen M., Yang-Fen T. L., P'eng F. K., Chou C. K. (1989). Transcripts of the insulin-like growth factors I and II in human hepatoma.. Cancer Res.

[OCR_01906] Talavera F., Reynolds R. K., Roberts J. A., Menon K. M. (1990). Insulin-like growth factor I receptors in normal and neoplastic human endometrium.. Cancer Res.

[OCR_01911] Thompson M. A., Cox A. J., Whitehead R. H., Jonas H. A. (1990). Autocrine regulation of human tumor cell proliferation by insulin-like growth factor II: an in-vitro model.. Endocrinology.

[OCR_01917] Tricoli J. V., Rall L. B., Karakousis C. P., Herrera L., Petrelli N. J., Bell G. I., Shows T. B. (1986). Enhanced levels of insulin-like growth factor messenger RNA in human colon carcinomas and liposarcomas.. Cancer Res.

[OCR_01921] Ullrich A., Gray A., Tam A. W., Yang-Feng T., Tsubokawa M., Collins C., Henzel W., Le Bon T., Kathuria S., Chen E. (1986). Insulin-like growth factor I receptor primary structure: comparison with insulin receptor suggests structural determinants that define functional specificity.. EMBO J.

[OCR_01940] Van Schravendijk C. F., Foriers A., Van den Brande J. L., Pipeleers D. G. (1987). Evidence for the presence of type I insulin-like growth factor receptors on rat pancreatic A and B cells.. Endocrinology.

[OCR_01948] Van Wyk J. J., Graves D. C., Casella S. J., Jacobs S. (1985). Evidence from monoclonal antibody studies that insulin stimulates deoxyribonucleic acid synthesis through the type I somatomedin receptor.. J Clin Endocrinol Metab.

[OCR_01954] Verland S., Gammeltoft S. (1989). Functional receptors for insulin-like growth factors I and II in rat thymocytes and mouse thymoma cells.. Mol Cell Endocrinol.

[OCR_01975] WIEDEMANN H. R. (1964). COMPLEXE MALFORMATIF FAMILIAL AVEC HERNIE OMBILICALE ET MACROGLOSSIE--UN "SYNDROME NOUVEAU"?. J Genet Hum.

[OCR_01959] Weima S. M., Stet L. H., van Rooijen M. A., van Buul-Offers S. C., van Zoelen E. J., de Laat S. W., Mummery C. L. (1989). Human teratocarcinoma cells express functional insulin-like growth factor I receptors.. Exp Cell Res.

[OCR_01964] Whitehead R. H., Novak U., Thomas R. J., Lukeis R. E., Walker F. E., Jones J. (1989). A new gastric carcinoma cell line (LIM1839) derived from a young Caucasian male.. Int J Cancer.

[OCR_01970] Widmer U., Zapf J., Froesch E. R. (1982). Is extrapancreatic tumor hypoglycemia associated with elevated levels of insulin-like growth factor II?. J Clin Endocrinol Metab.

[OCR_01986] Williams D. W., Williams E. D., Wynford-Thomas D. (1989). Evidence for autocrine production of IGF-1 in human thyroid adenomas.. Mol Cell Endocrinol.

[OCR_01980] Willison K. (1991). Opposite imprinting of the mouse Igf2 and Igf2r genes.. Trends Genet.

[OCR_01989] Yashiro T., Ohba Y., Murakami H., Obara T., Tsushima T., Fujimoto Y., Shizume K., Ito K. (1989). Expression of insulin-like growth factor receptors in primary human thyroid neoplasms.. Acta Endocrinol (Copenh).

[OCR_01996] Yee D., Paik S., Lebovic G. S., Marcus R. R., Favoni R. E., Cullen K. J., Lippman M. E., Rosen N. (1989). Analysis of insulin-like growth factor I gene expression in malignancy: evidence for a paracrine role in human breast cancer.. Mol Endocrinol.

[OCR_02012] Zapf J., Froesch E. R. (1986). Insulin-like growth factors/somatomedins: structure, secretion, biological actions and physiological role.. Horm Res.

[OCR_01932] van Buul-Offers S., Ueda I., Van den Brande J. L. (1986). Biosynthetic somatomedin C (SM-C/IGF-I) increases the length and weight of Snell dwarf mice.. Pediatr Res.

